# Barriers and Opportunities for Implementation of Outcome-Based Spread Payments for High-Cost, One-Shot Curative Therapies

**DOI:** 10.3389/fphar.2020.594446

**Published:** 2020-12-08

**Authors:** Sissel Michelsen, Salma Nachi, Walter Van Dyck, Steven Simoens, Isabelle Huys

**Affiliations:** ^1^ Clinical Pharmacology and Pharmacotherapy, KU Leuven, Leuven, Belgium; ^2^ Healthcare Management Centre, Vlerick Business School, Ghent, Belgium

**Keywords:** advanced therapy medicinal product, curative therapy, spread payment, outcome-based agreement, affordability, pay-for-performance, annuity, managed entry agreement

## Abstract

**Background:** The challenging market access of high-cost one-time curative therapies has inspired the development of alternative reimbursement structures, such as outcome-based spread payments, to mitigate their unaffordability and answer remaining uncertainties. This study aimed to provide a broad overview of barriers and possible opportunities for the practical implementation of outcome-based spread payments for the reimbursement of one-shot therapies in European healthcare systems.

**Methods:** A systematic literature review was performed investigating published literature and publicly available documents to identify barriers and implementation opportunities for both spreading payments and for implementing outcome-based agreements. Data was analyzed via qualitative content analysis by extracting data with a reporting template.

**Results:** A total of 1,503 publications were screened and 174 were included. Main identified barriers for the implementation of spread payments are reaching an agreement on financial terms while considering 12-months budget cycles and the possible violation of corresponding international accounting rules. Furthermore, outcome correction of payments is currently hindered by the need for additional data collection, the lack of clear governance structures and the resulting administrative burden and cost. The use of spread payments adjusted by population- or individual-level data collected within automated registries and overseen by a governance committee and external advisory board may alleviate several barriers and may support the reimbursement of highly innovative therapies.

**Conclusion:** High-cost advanced therapy medicinal products pose a substantial affordability challenge on healthcare systems worldwide. Outcome-based spread payments may mitigate the initial budget impact and alleviate existing uncertainties; however, their effective implementation still faces several barriers and will be facilitated by realizing the required organizational changes.

## Introduction

The increased development of gene therapy medicinal products (GTMPs) delivers the promise of therapies with long-term, possibly curative, benefits after a one-time administration (EUR-Lex website, 2007). To date, six one-shot gene therapies have received market authorisation by the European Commission (Hanna et al., 2017; Ginn et al., 2018). However, market uptake of these therapies has been limited in Europe due to difficulties in obtaining reimbursement. On average four gene therapies are reimbursed in France, Germany, Italy and the United Kingdom while their uptake is lacking in other European countries (Alliance for Regenerative Medicine, 2019). This limited uptake is partially explained by the high prices set by manufacturers possibly creating a threat to the sustainability of the healthcare budget in case it would need to absorb potential peaks of treatment prices of €320,000 for CAR-T cell therapies up to €1,900,000 for gene therapy treating spinal muscular atrophy (Touchot and Flume, 2017; AveXis, 2019). Therefore, payers may experience difficulty with ensuring that the therapy is still affordable given the healthcare budget especially limiting market access in lower income countries (Hanna et al., 2017). Additionally, payers need to be convinced of their added value while experiencing decision-uncertainty due to persisting uncertainties caused by shortcomings in clinical trial design and the unavailability of long-term data on efficacy and safety (Carr and Bradshaw, 2016; Hanna et al., 2017).

Standard reimbursement systems are currently based on up-front, single payments from annual governmental budgets and access to high-cost treatments with substantial uncertainty is enabled by the use of managed entry agreements (MEAs). Moreover, outcome-based agreements (OBA) are used to provide access to therapies with uncertain clinical benefits by adapting the amount or level of reimbursement based on achieved health outcomes (Garrison Jr et al., 2013; Drummond, 2015; Carlson et al., 2017). Next to the existing reimbursement systems, payers may consider alternative reimbursement structures to mitigate both the unaffordability and the uncertainties on real-world benefits of high-cost, one-shot therapies (Carr and Bradshaw, 2016; Annemans and Pani, 2017; Marsden et al., 2017; Kefalas et al., 2018; Jorgensen et al., 2019; AMCP, 2019; FoCUS, 2019b). One of the more frequently cited novel reimbursement structures is paying for gene therapies with instalments over multiple years corrected for real-world outcomes of the treatment, otherwise called outcome-based spread payments. Annuity payments, a specific form of spread payments, are paid once every year instead of every few months (Garrison Jr et al., 2013; Edlin et al., 2014; Carr and Bradshaw, 2016; Marsden et al., 2017; Jorgensen and Kefalas, 2017; Hettle et al., 2017; Kefalas et al., 2018; FoCUS, 2019b). This reimbursement method combines an OBA with spread payments over time which may solve the immediate unaffordable budget impact caused by the high upfront treatment price while the inclusion of an OBA foresees the correction of payments for real-world performance solving both short- and long-term clinical uncertainties (Jorgensen and Kefalas, 2017; Yeung et al., 2017; Faulkner et al., 2018; Schaffer et al., 2018; Jönsson et al., 2019; Towse and Fenwick, 2019).

Even though this payment structure has been widely discussed as a possible solution for the sustainable reimbursement of high-cost, one-shot therapies, implementation of outcome-based spread payments within European healthcare systems is still limited due to practical difficulties experienced by payers, developers and healthcare providers (i.e., hospitals) (Carlson et al., 2017; Hanna et al., 2018; Barlow et al., 2019). Therefore, the aim of this study is to provide a broad overview for European payers, manufacturers and healthcare providers of all barriers and potential opportunities for the implementation of outcome-based spread payments in single-payer (national) healthcare systems. This study investigated barriers related to the implementation of spread payments focusing on their organisation and possible legislative hurdles. Furthermore, barriers were investigated that are caused by difficulties in correcting payments for outcomes focusing on the process of data collection and the required governance structures. This overview is of added value to policy- and decision-makers who wish to implement such novel reimbursement structures in European single-payer healthcare systems and may assist developers who aim to propose this payment structure for reimbursement of their innovative products.

## Methods

A systematic literature review was performed of gray literature and peer-reviewed articles published in embase and Pubmed to identify all relevant barriers for implementation of outcome-based spread payments. Barriers for implementation of outcome-based spread payments may arise through either the notion of spreading payments or the required outcome correction of payments. Therefore, the systematic search aimed to identify records that discussed barriers for implementation of OBAs (with upfront or spread payments) and/or implementation of spread payments. OBAs were defined according to the definition formulated by Garrison et al.: “a plan by which the performance of the product is tracked in a defined patient population over a specified period of time and the level or continuation of reimbursement is based on the health and economic outcomes achieved” (Garrison Jr et al., 2013). This definition includes both coverage with evidence development (CED) schemes and performance-linked reimbursement schemes using a moneyback or outcomes guarantee. To ensure a complete overview, barriers were identified for CED and performance-linked reimbursement schemes that could be relevant for the implementation of OBAs in combination with spreading payments over time. No uniform definition exists yet for reimbursement with spread payments. Therefore, we defined spread payments as: “Replacing one-time up-front payment by a stream of payments spread over time with/without correction of continued payments for achieved outcomes” (Edlin et al., 2014). Based on the abovementioned definitions, a search strategy was launched on November 2019 focusing on managed entry, outcome correction and spreading payments (Supplementary Table S1). No MeSH/Emtree terms were used since no specialised terms on managed entry in the context of reimbursement exist yet. Gray literature was collected through handsearching and includes publicly available documents from payers, health technology assessment bodies, research institutions and multi-stakeholder initiatives.

After retrieval of all records and removal of duplicates, titles and abstracts were independently screened by two researchers (SM and SN). Articles were considered for inclusion if (EUR-Lex Website, 2007) the article was written in English (Hanna et al., 2017), the record was a conference abstract or the full text was available (Ginn et al., 2018), the record discussed barriers for the implementation of OBAs with upfront or spread payments (Alliance for Regenerative Medicine, 2019), the record discussed barriers for implementation of spread payments and (Touchot and Flume, 2017) payment models were discussed in the context of pharmaceuticals reimbursement. This review focuses on barriers important for the European context of single-payer (national) healthcare systems. However, articles describing relevant barriers observed in non-European jurisdictions were also included to ensure a full overview. Abstracts were included for full-text review if one of the two researchers judged the article to comply with all inclusion criteria. Full text articles, conference abstracts and gray literature were screened by one researcher (SM) using the same inclusion and exclusion criteria and additional records were included by screening the reference lists of included articles (snowballing). All types of records (conference abstracts, peer-reviewed articles and gray literature) were included to allow for a complete overview of all mentioned barriers for outcome-based spread payments. Data was analyzed via qualitative content analysis (Finfgeld-Connett, 2013) by extracting data in broad categories via a reporting template: a) agreement type b) type of therapy c) geographical scope d) all cited barriers e) payment modality f) legislative requirements g) collection of data and h) governance structure.

## Results

**FIGURE 1:**
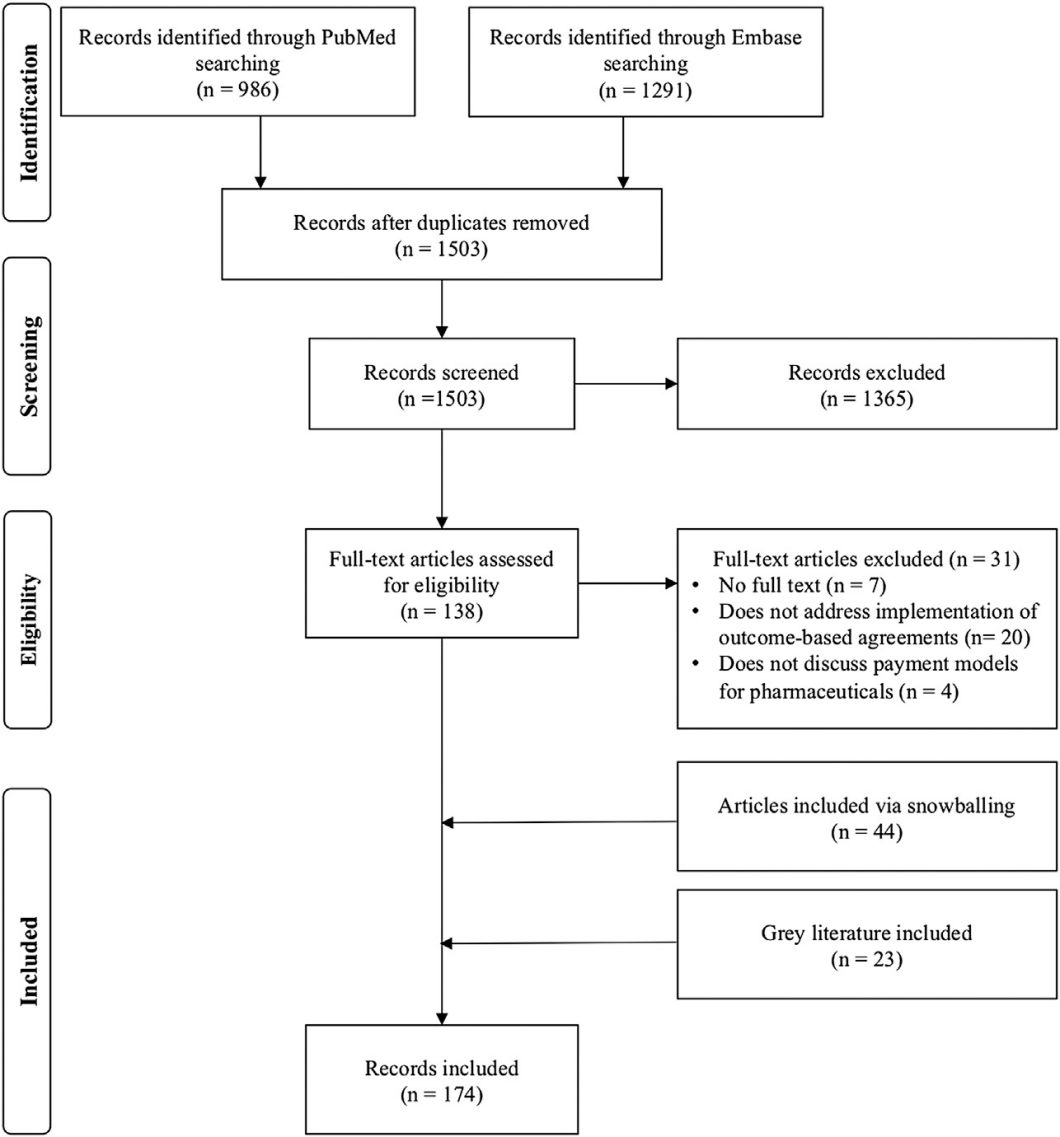
PRISMA diagram of the literature search and inclusion of publications.

One thousand five hundred three records were extracted from the PubMed and embase databases after removal of duplicates. After title/abstract screening, 1,365 records were excluded and 138 full text articles were assessed. After snowballing and assessing gray literature, 174 records were included for analysis (Figure 1). Of the identified records, 41 publications discuss barriers for the use of outcome-based spread payments, 38 publications for spread payments without outcome correction and 155 articles address barriers for implementation of upfront payments with outcome correction (Supplementary Table S2).

Barriers for spreading payments were identified from records discussing spread payments with/without outcome correction. On the other hand, barriers for correcting payments for outcomes were identified from publications discussing outcome correction of upfront and/or spread payments. An overview of all identified barriers and proposed opportunities can be found in Table 1.

**TABLE 1:** Categories for which barriers and proposed opportunities for the implementation of spread payments and the outcome correction of payments were identified.

	Category	Barriers	Potential opportunities
Spread payments	Payment structure	Determine spread payment amounts	NR
		Determine optimal duration of payments	Payment as long as therapy delivers benefit
			Payments as long as the patient is alive
	Organisation of payments	Conflicting financial flows between stakeholders	Direct purchasing of therapy by payer
		12-months budget cycles	Adapt European accounting rules
			Adapt national accounting rules
		Build agreements reflecting competitive environment	Horizon scanning
			Re-opener clauses of agreement after entry of competitive product
			Pre-agreed adjustment of payment based on expected entry of competitive product
Outcome correction of payments	Outcome correction	Correcting payments for real-world performance	Individual-level payment adjustment
			Population-based payment adjustment
	Process of data collection	Determining study design	Value of information analysis
			Build disease registries with standardized data elements covering the continuity of care
		Selecting appropriate outcomes	Build minimal core outcome set
			Use validated surrogate endpoints
			Reach multi-stakeholder agreement on definition of treatment success
		Data quality and analysis	Training of healthcare professionals, manufacturers and payers on analysis and interpretation of results
			Monitoring of data collection with yearly data audits
	Organisation of data collection	High burden of data collection	Use administrative and claims databases
			Cross-country collaboration by coordinating multi-country data collection
		Insufficient data infrastructure	Automation of data collection
			Integration and interoperability of collection systems
		Ensure personal data protection	Sharing of aggregated, population-based data
			High-quality risk management
	Governance	Insufficient governance structure	Define stakeholders' roles and responsibilities
			Define stakeholders' interest and incentives
			Define clear funding arrangements
			Determine complete data collection process
			Determine structure to initiate payments
			Build steering committee and external advisory board
		Administrative burden and cost	Implement a streamlined governance structure
			Initial investment in high-quality IT infrastructure
			Continued investment in the education and support of experienced staff

NR, Not reported.

### Barriers and Potential Opportunities for Implementing Spread Payments

Multiple authors emphasized that multi-stakeholder agreement between payers and developers on financial terms of the MEA is crucial to enable the use of spread payments (Brennan and Wilson, 2014; Edlin et al., 2014; Philipson, 2014; Basu, 2015; Drummond, 2015; Touchot and Flume, 2015; Jorgensen and Kefalas, 2015; Kleinke and McGee, 2015; Carr and Bradshaw, 2016; Montazerhodjat et al., 2016; Carlson et al., 2017; Marsden et al., 2017; Jorgensen and Kefalas, 2017; Yeung et al., 2017; Value in Health, 2017; Carr et al., 2018; Faulkner et al., 2018; Hanna et al., 2018; Hampson et al., 2018; Lidonnici et al., 2018; Sachs et al., 2018; Senior, 2018; Tuffaha and Scuffham, 2018; Salzman et al., 2018; Schaffer et al., 2018; AMCP, 2019; Drummond et al., 2019; Jönsson et al., 2019; FoCUS, 2019a; FoCUS, 2019b; Infante et al., 2019; Maes et al., 2019; Towse and Fenwick, 2019). However, many uncertainties on the ideal duration of spread payments, payment amount per installment and payment linkage to outcomes remain. Several publications argue to spread payments over the duration of benefit or effect of the therapy (Edlin et al., 2014; Hettle et al., 2017; Jorgensen and Kefalas, 2017; Jönsson et al., 2019; Maes et al., 2019) with a time limit on the payment period (2–5 years) (Faulkner et al., 2018; Hanna et al., 2018; Maes et al., 2019), while others argue for continued payments as long as the patient is alive (Schaffer et al., 2018; Towse and Fenwick, 2019). Although several recommendations exist, no article yet describes a formal method to determine the optimal duration of such spread payments. Furthermore, the CAR-T mock appraisal performed by the National Institute for Health and Care Excellence (NICE) and the ICER Policy Summit indicated that payers require clarity on the potential price-increasing effect of spreading payments due to the interest rate required by the manufacturer on deferred payments (Hettle et al., 2017; Hampson et al., 2018; Maes et al., 2019).

Besides difficulties with reaching agreement on financial terms of spread payments, current financial flows between stakeholders and annual budget cycles could hinder the operationalization of spread payments (Brennan and Wilson, 2014; Kleinke and McGee, 2015; Touchot and Flume, 2015; Montazerhodjat et al., 2016; Proach et al., 2016; Carlson et al., 2017; Marsden et al., 2017; Jorgensen and Kefalas, 2017; Spark et al., 2017; Yeung et al., 2017; Faulkner et al., 2018; Hampson et al., 2018; Hanna et al., 2018; Nevins et al., 2018; Sachs et al., 2018; Salzman et al., 2018; Schaffer et al., 2018; Senior, 2018; Tuffaha and Scuffham, 2018; Alliance for Regenerative Medicine, 2019; FoCUS, 2019a; FoCUS, 2019b; Jorgensen et al., 2019; AMCP, 2019; Towse and Fenwick, 2019; Barlow et al., 2019; Drummond et al., 2019; Infante et al., 2019; Maes et al., 2019). In Europe, therapies are mainly purchased by healthcare providers and subsequently reimbursed by the responsible payer. However, spreading payments may conflict with standard financial flows since a link is needed between the medical system and the financial system to allow the initiation of payments when outcomes are achieved and payments have to be tracked over multiple years which may create additional administrative costs (Garrison Jr et al., 2013; Kleinke and McGee, 2015; Slocomb et al., 2017; Richardson and Ling, 2018). These difficulties are similar to the financial challenges experienced by OBAs with upfront payments, such as the OBA for sunitinib in the United Kingdom, where the healthcare provider may need to adjust stock control systems, verify if the cost of the drug is correctly reflected in the financial systems and ensure payments are correctly triggered (Carlson et al., 2009; Lucas et al., 2009; Williamson and Thomson, 2010; Espin et al., 2011; Coulton et al., 2012; Towse et al., 2012; Ferrario and Kanavos, 2013; Navarria et al., 2015; Faulkner et al., 2016; Gerkens et al., 2017; Pauwels et al., 2017; Toumi et al., 2017; Bouvy et al., 2018; Cole et al., 2019; Mahendraratnam et al., 2019; Makady et al., 2019; Wenzl and Chapman, 2019). To avoid the burden of current financial flows, payers could directly purchase the therapeutic product from the manufacturer and distribute it to the healthcare provider, as proposed by Spark Therapeutics for the reimbursement of Luxturna (Senior, 2018). This would enable payers to alleviate the burden on healthcare provider budgets from purchasing high-cost medicines and therefore eliminate the buy-and-bill inventory risk. However, direct purchasing may result in loss of mark-ups enjoyed by the healthcare provider and thus disrupt provider revenue (FoCUS, 2019b; Barlow et al., 2019).

Spreading payments over multiple years may conflict with the standard 12-months financial cycles of both the payer and manufacturer (Edlin et al., 2014; Faulkner et al., 2016; Thompson et al., 2016; Danzon, 2018; Faulkner et al., 2018; AMCP, 2019; Jorgensen and Kefalas, 2019). Payer’s yearly budgets may not be equipped to implement spread payments over time since the complete cost of the one-shot therapy will have to be budgeted in the year of administration (Touchot and Flume, 2015; Faulkner et al., 2016; Schaffer et al., 2018; Alliance for Regenerative Medicine, 2019). Similarly, Nevins et al. observed that manufacturers prefer predictable revenue streams, have to account for the cost of credit and need to consider their own financial obligations (Hettle et al., 2017; Nevins et al., 2018). Therefore, a change in accounting standards is required to budget treatments over multiple years (Gottlieb and Carino, 2014; Hettle et al., 2017; FoCUS, 2019a; FoCUS, 2019b; Maes et al., 2019). However, changing accounting standards will be complicated by required compliance to existing national and European accounting rules (Gottlieb and Carino, 2014; Slocomb et al., 2017; Alliance for Regenerative Medicine, 2019; Cole et al., 2019; FoCUS, 2019a; FoCUS, 2019b; Maes et al., 2019). According to the European System of Accounts (ESA), the full cost will have to be budgeted within the year of treatment administration which means that payments in consecutive years will be defined as a loan and will subsequently increase government debt (Maes et al., 2019). Therefore, the European accounting rules may nullify the budgetary advantage of spreading payments over time. Maes et al. propose two possible solutions to comply with the ESA: the payer pays for the service of the treatment delivering long-term health outcomes or the payer pays for data services as a delivered data package per year instead of paying for a single treatment administration (Maes et al., 2019). Furthermore, healthcare systems wishing to implement spread payments may face national legal barriers which may require adjustment of country-specific regulations (Carlson et al., 2009; Carlson et al., 2011; Espin et al., 2011; Ferrario and Kanavos, 2013; Tuna et al., 2014; Kleinke and McGee, 2015; Barlas, 2016b; Proach et al., 2016; Montazerhodjat et al., 2016; Kanavos et al., 2017; Nazareth et al., 2017; NEHI, 2017; PWC Health Research Institute, 2017; Spark et al., 2017; Goncalves et al., 2018; Salzman et al., 2018; Infante et al., 2019; Lorente et al., 2019; Mahendraratnam et al., 2019). For instance, the current legislation in Spain only allows long-term spending for certain investments which excludes medicines and Sweden does not allow payments for more than three years for non-investment consumables (that are not purchased to deliver financial return) (Alliance for Regenerative Medicine, 2019).

Lastly, during agreements with spread payments over multiple years, payers could consider the possibility that products lose their exclusivity during the course of the agreement or new, possibly better, products enter the market (Hutton et al., 2007; Adamski et al., 2010; Menon et al., 2011; Goodman et al., 2019). Agreements that do not consider market dynamics may reduce the relevance of the evidence generated (Pauwels et al., 2017; Makady et al., 2019) and may disincentivise the development of new technologies since conditions stipulated within current agreements may influence future developers (Trueman et al., 2010; Tuffaha and Scuffham, 2018). Studies assessing experiences with OBAs emphasized that horizon scanning is crucial to anticipate novel products to inform whether a long-term contract should be concluded or which conditions should be captured within the contract to subject the innovative therapy to the effects of market competition (Menon et al., 2011; Pauwels et al., 2017; AMCP, 2019; Wenzl and Chapman, 2019). These conditions could either be a pre-agreed adjustment of the payment in expectation of competition, as proposed by Towse et al. investigating the implications of paying for one-shot cures vs. repeat administrations. Contrarily, Schaffer et al. propose the addition of re-opener clauses that foresee provisions for payment adjustment whereas an evaluation of Belgian MEAs suggested the complete termination of the agreement when exclusivity rights expire or a competitive product enters the market (Kanavos et al., 2017; Nazareth et al., 2017; AMCP, 2019; Infante et al., 2019).

### Barriers and Potential Opportunities for Correcting Payments for Achieved Real-World Outcomes

Spreading payments over time may mitigate the substantial budget impact caused by high-cost, one-shot curative therapies. However, to reduce uncertainties on both efficacy and safety, payments need to be corrected for outcomes achieved in the real-world. Therefore, spread payments for the reimbursement of medicines can be implemented as an OBA to correct payments for real-world performance. Nevertheless, to enable the implementation of OBAs to correct payments for achieved outcomes several hurdles will have to be overcome.

#### Correcting Payments for Achieved Real-World Outcomes

Spread payments have not yet been widely implemented in practice but several authors propose modalities to correct payments for real-world performance. However, ambiguity exists on how payments should be linked to outcomes and several forms of outcome correction have been described either based on individual or population-level data. For spread payments, outcome correction could be based on individual patient data by completely terminating all payments after treatment failure within the individual patient (Edlin et al., 2014; Kleinke and McGee, 2015; Carlson et al., 2017; Jorgensen and Kefalas, 2017; Hettle et al., 2017; Faulkner et al., 2018; Hampson et al., 2018; Oren and Oren, 2018; Schaffer et al., 2018; FoCUS, 2019a; FoCUS, 2019b; Towse and Fenwick, 2019). Outcome correction via individual patient data enables real-time adjustment of payments (Annemans and Pani, 2017; Fox and Watrous, 2017) but could stimulate payers or manufacturers to adversely select high or low risk patients to influence payment amounts (Garrison Jr et al., 2015; Maes et al., 2019). Furthermore, individual patient outcome is dependent on the correct administration of the treatment (Edlin et al., 2014) and individual performance is not useful to verify real-world effectiveness of the treatment for the complete patient population (Launois et al., 2014; Wenzl a Chapman, 2019). Contrarily, payments could be adjusted based on population-level data where the payment for all patients is adjusted post-hoc if aggregated outcomes do not meet a predefined target (Garrison Jr et al., 2013; Van De Vijver et al., 2016; Yeung et al., 2017; Cole et al., 2019; Maes et al., 2019). However, ambiguity remains whether the whole patient population or a sample of patients should be tracked, how missing data should be handled and whether this is feasible for small patient populations (AMCP, 2017; FoCUS, 2019b). Furthermore, several possibilities exist for the adjustment of payment amounts based on population-based outcomes. Payments could be adapted binary with a fixed decrease in payment if patient responses drop below a certain threshold, in a stepped manner where the payment amount can have different levels based on the outcomes achieved or in a continuous manner where the payment is a function of the outcomes measured (Cole et al., 2019; FoCUS, 2019b). Furthermore, an increase in payment amount when the therapy performs better than expected could reward manufacturers and incentivize the development of highly effective therapies (Annemans and Pani, 2017; Hettle et al., 2017; Cole et al., 2019). However, an increase of payment amounts due to better performance and thus a total price increase is currently not possible due to international reference pricing and national legislations in a European context (Annemans and Pani, 2017; Tuffaha and Scuffham, 2018; Cole et al., 2019).

#### The Organization of Data Collection

Organising and performing data collection are two of the most frequently discussed barriers for implementation of OBAs for payers, developers and healthcare providers (Sudlow and Counsell, 2003; de Pouvourville, 2006; Carlson et al., 2010; McCabe et al., 2010; Raftery, 2010; Stafinski et al., 2010; Williamson, 2010; Jaroslawski and Toumi, 2011b; Klemp et al., 2011; Neumann et al., 2011; Cascade et al., 2012; Goldenberg and Bachman, 2012; Xoxi et al., 2012; Bibeau et al., 2014; Gibson and Lemmens, 2014; Li et al., 2014; Garattini et al., 2015; Lu et al., 2015; Lucas and Wong, 2015; Mohseninejad et al., 2015; Barlas, 2016b; Carr and Bradshaw, 2016; Malik, 2016; Pouwels et al., 2016; van de Wetering et al., 2017; Duhig et al., 2018; Ernst and Young, 2018a; Ernst and Young, 2018b; EXPH, 2018; Goldenberg et al., 2018; Jorgensen et al., 2018; Stirnadel-Farrant et al., 2018; Urbinati et al., 2018; Federici et al., 2019; Macaulay and Turkstra, 2019; Mundy et al., 2019; Pace et al., 2019; Kannarkat et al., 2020). First, experiences with OBAs in the Netherlands and the United Kingdom highlight that payers should perform a value of information analysis to guide the decision to engage in an OBA to confirm that the benefits from additional evidence collection are higher than the cost of collecting the data (Ferrario and Kanavos, 2013; Pauwels et al., 2017; Makady et al., 2019; Towse and Fenwick, 2019). When the value of information is high, the selection of a fitting study design should ensure the gathering of high-quality data that alleviates remaining uncertainties relevant to the payer (Theunissen et al., 2010; Towse and Garrison Jr, 2010; Vitry and Roughead, 2014; Gerkens et al., 2017). However, uncertainty exists on which evidence is needed, which outcomes should be selected and from which sources the data should be gathered (Ferrario and Kanavos, 2013; AMCP, 2017). To enable the collection of population- and individual-based data, the use of registries as prospective observational study design has been recommended (Theunissen et al., 2010; Towse and Garrison Jr, 2010; Menon et al., 2011; Vitry and Roughead, 2014; Pritchett et al., 2015; Schmetz et al., 2018; Jönsson et al., 2019). An expert panel reviewing the applicability of current HTA practices to the needs of ATMPs and the evaluation of MEAs by the Belgian healthcare knowledge center recommend registries to consist of standardized data elements and cover the continuity of care, including the primary care setting for possibly cured patients, by being linkable to other databases resulting in the provision of accurate, reliable and complete information (Towse and Garrison Jr, 2010; Ferrario and Kanavos, 2013; Cole et al., 2019).

Next to the set-up of registries, several publications report that payers and developers face difficulties to select appropriate outcomes as decision-rule for payment adjustment based on treatment performance (Breckenridge and Walley, 2008; Goldenberg and Bachman, 2012; Kocsis et al., 2015; Abou-El-Enein et al., 2016; Hettle et al., 2017; Holleman et al., 2017; Hanna et al., 2018; Pham and Carlson, 2018; Sandhu and Heidenreich, 2018; Jönsson et al., 2019; Mahendraratnam et al., 2019). Accessible and easily measurable outcomes in the short-term to medium-term which are clinically relevant, useful and important to all stakeholders are recommended (Dankó et al., 2009; Kiernan, 2016; Pouwels et al., 2016; Goble et al., 2017; Kazi et al., 2017; Yeung et al., 2017; Danzon, 2018; EXPH, 2018; Cole et al., 2019). A minimal core outcome set per disease could be built such as survival, disease progression, relapse or recurrence, long-term side effects and return to normal activities for oncological diseases as proposed by Cole et al. (Cole et al., 2019) or disease progression, unacceptable toxicity not allowing continuation of treatment and toxicity-related death as used by the national health service in Italy (Garattini and Casadei, 2011). However, payers are faced with the fact that effects of possibly curative therapies might only appear in the long-term while actionable outcomes are restricted to those measured in the short-term which is similar to the experienced difficulties of the United Kingdom MS scheme to provide conclusive answers on long-term functional outcomes (Lage et al., 2013; Carr and Bradshaw, 2016; Kiernan, 2016; Seeley and Kesselheim, 2017). This would require the use of surrogate endpoints which may give false reassurance of performance (Hettle et al., 2017; Pauwels et al., 2017; Seeley and Kesselheim, 2017; Toumi et al., 2017). Therefore, validated surrogate endpoints that are proven predictors of hard clinical endpoints and uninfluenced by other treatments should be selected (Garber and McClellan, 2007; McCabe et al., 2010; Brennan and Wilson, 2014; Franken et al., 2014; Launois et al., 2014; Carr and Bradshaw, 2016; Gerkens et al., 2017; Toumi et al., 2017; Yeung et al., 2017). However, proven validated surrogate endpoints may not exist for all disease areas which complicates agreement between payer and developer on the chosen outcome. Furthermore, multi-stakeholder agreement on the definition of success, by determining the baseline and target performance, has shown to be difficult but is crucial to define the link between the achieved outcomes and payment (McCabe et al., 2010; Neumann et al., 2011; Towse et al., 2012; Marsden et al., 2017; Jönsson et al., 2019).

Even when the ideal study set-up could be reached and the optimal outcome is selected, data collection is dependent on intensive human and financial resources from the healthcare provider and payer. Administrative and claims databases, as already used in six of twelve countries using OBAs interviewed by OECD (Wenzl and Chapman, 2019), might offer an opportunity for low-burden data collection by measuring hospitalizations and alternative drug interventions as surrogate endpoints for disease progression (Garrison Jr et al., 2015; Yu et al., 2017; Robinson et al., 2018; Schaffer et al., 2018; Makady et al., 2019; Wenzl and Chapman, 2019). However, these data do not provide detailed information on clinical outcomes which might limit their usefulness (Garrison Jr et al., 2015; Seeley and Kesselheim, 2017). Another option to increase data collection capabilities in Europe is enabling cross-country collaboration by coordinating multi-country clinical data collection with interoperable registries to reduce the burden of data collection and avoid duplication of collection efforts (Towse and Garrison Jr, 2010; Ferrario and Kanavos, 2013; Morel et al., 2013; Marsden et al., 2017; Jorgensen and Kefalas, 2017; Kanavos et al., 2017; Bouvy et al., 2018; Alliance for Regenerative Medicine, 2019; Jorgensen et al., 2019; Jorgensen and Kefalas, 2019; Maes et al., 2019). This collaboration could be organised by aligning the evidence needed for follow-up requirements of the European Medicines Agency (EMA) with identical requests for post-reimbursement evidence by several national payers (Ferrario and Kanavos, 2013; Marsden et al., 2017; Gerkens et al., 2017; Jorgensen and Kefalas, 2017; Geldof et al., 2019; Jorgensen et al., 2019; Maes et al., 2019). A first step toward international collaboration has been made by the EMA that will develop the Data Analysis Real World Interrogation Network (DARWIN) to access and analyze healthcare data from across the EU (Hines et al., 2020). Currently, only the Italian national health service possesses a national registry for both regulatory and reimbursement purposes and joint scientific advice will be needed from EMA and the different European health technology assessment bodies to enable collaboration between the regulatory agency and multiple national payers (Bouvy et al., 2018; Jorgensen and Kefalas, 2019).

The collection of detailed clinical data will require the set-up of specific data collection systems or the advanced extraction of data from electronic medical records (Lewis et al., 2015; Goble et al., 2017; Yeung et al., 2017; Wenzl and Chapman, 2019). The presence of a robust data infrastructure requires the establishment of data systems that operate in an automated manner and are virtually connected to integrate all existing information systems within common data formats (Garrison Jr et al., 2015; Gerkens et al., 2017; NEHI, 2017; Goncalves et al., 2018; Maes et al., 2019; Makady et al., 2019). Automation of data collection efforts will require the extensive use of electronic medical records which may be converted into an analysable form (Seeley and Kesselheim, 2017; Robinson et al., 2018; Maes et al., 2019) and the integration and interoperability of data systems that link clinical and financial data (Value in Health, 2017; Antonanzas et al., 2019; Jorgensen et al., 2019; Mahendraratnam et al., 2019). The Italian web-based monitoring registries are currently the most frequently cited as good practice serving as a complete post-marketing surveillance system to share information between health authorities, clinicians, pharmacists and payers with automated procedures and analysis (Carlson et al., 2011; Garrison Jr et al., 2013; De Rosa et al., 2015; Garattini et al., 2015; Montilla et al., 2015; Pauwels et al., 2017; Maskineh and Nasser, 2018; Jorgensen et al., 2019). Other European countries, such as Belgium, Spain and the United Kingdom, highlight the importance of leveraging existing databases to collect both clinical and budgetary data with possible infrastructure upgrades or including new organisational circuits to avoid delays in obtaining data (Clopes et al., 2017; Gerkens et al., 2017; Bouvy et al., 2018; Kefalas et al., 2018; Jorgensen et al., 2019). Jörgensen et al. have calculated the cost estimate for upgrading the current oncological data collection infrastructure via manual workaround or via automation to enable outcome-based reimbursement in the United Kingdom. The analysis showed that in both cases the upfront cost would be mainly caused by the technological upgrade and would be five times higher to allow automation. However, the high annual costs for the manual workaround are substantially lower when the infrastructure is automated and will not be sensitive to the number of contracts (Jorgensen and Kefalas, 2019). Another option is mimicking the Italian system by building a national registry independent of disease area which would lower the cost per MEA set-up but would require a substantial initial investment (Kefalas et al., 2018; Jorgensen and Kefalas, 2019).

Based on experiences with OBAs in Europe, the incomparability of patients, missing data and the presence of confounders pose threats to data quality which may complicate the analysis and interpretation of evidence to support the adjustment of spread payments (Boggild et al., 2009; Adamski et al., 2010; Lage et al., 2013; Van Der Meijden et al., 2013; Vitry and Roughead, 2014; Franken et al., 2014; Garrison Jr et al., 2015; Touchot and Flume, 2015; Carroll and Truglio, 2016; Value in Health, 2017; Kim et al., 2018; Sachs et al., 2018; Tuffaha and Scuffham, 2018; Cole et al., 2019). Furthermore, studies investigating payer opinions showed that low data quality may cause skepticism of payers toward real-world evidence possibly influencing their willingness to use OBAs (Carr et al., 2018; Makady et al., 2019). Additionally, gathering high-quality data is dependent on the input of data from healthcare professionals which was cited as insufficient during CED programs in the Netherlands and during OBAs in Italy due to low compliance with registry procedures (Garattini and Casadei, 2011; Jaroslawski and Toumi, 2011b; Pauwels et al., 2017; Bouvy et al., 2018). Therefore, Makady et al. recommend to invest in the extensive training of healthcare professionals, manufacturers and payers on analysis and interpretation of the results for OBAs (Makady et al., 2019). Furthermore, progress of data collection could be monitored by frequent payer audits to correct for errors during the course of the agreement (Makady et al., 2019; Makady et al., 2019) and to verify if the registry is providing useful information (Mohseninejad et al., 2015; Annemans and Pani, 2017; Gerkens et al., 2017; FoCUS, 2019b).

Lastly, use of data collection systems to collect personal data will require compliance with data privacy regulations such as the General Data Protection Regulation (GDPR) to protect personal data during OBAs (Klemp et al., 2011; Coulton et al., 2012; Franken et al., 2014; Drummond, 2015; Barlas, 2016a; Proach et al., 2016; Van De Vijver et al., 2016; PWC Health Research Institute, 2017; van de Wetering et al., 2017; Duhig et al., 2018; Ernst and Young, 2018a; Ernst and Young, 2018b; EXPH, 2018; Mundy et al., 2019). According to the GDPR, patients may withdraw consent and withdraw their data from the holder files which may increase missing data in registries (Alliance for Regenerative Medicine, 2019; FoCUS, 2019b). Additionally, contractual terms of OBAs may require sharing of identifiable personal data to allow for adjustment of payments. Therefore, additional risk management may be needed to ensure that personal data protection remains guaranteed (Menner and Lewandowska, 2018; Maes et al., 2019) especially for agreements based on individual patient data where the need for identifiable patient data is higher than agreements dependent on aggregated population-level data (Gerkens et al., 2017). In both cases, correct provisions for data protection and sharing are needed with adequate safeguards determining which parties will have access to the data and in which form (Faulkner et al., 2016; Gerkens et al., 2017; Sachs et al., 2018; Jorgensen and Kefalas, 2019; FoCUS, 2019b; Maes et al., 2019).

#### The Implementation of a Governance Framework

High-quality governance of OBAs is crucial to support financial flows of the agreement, data collection and reinforcement of the relevant legislation. Therefore, several publications, discussing barriers from payer, developer and provider perspective, highlight the current lack of clear governance structures and recommend to build a framework that details every step of the process with specification of every stakeholders’ roles, responsibilities, interests and incentives (Carlson et al., 2009; Trueman et al., 2010; Ferrario et al., 2011; Jaroslawski and Toumi, 2011a; Jaroslawski and Toumi, 2011b; Neumann et al., 2011; Goldenberg and Bachman, 2012; Xoxi et al., 2012; Towse et al., 2012; Gottlieb and Carino, 2014; Li et al., 2014; Vitry and Roughead, 2014; Drummond, 2015; Lu et al., 2015; Lu et al., 2015; Lucas and Wong, 2015; Lucas and Wong, 2015; Polimeni et al., 2016; Calabrese et al., 2017; Value in Health, 2017; Slocomb et al., 2017; Toumi et al., 2017; Nazareth et al., 2017; NEHI, 2017; Seeley and Kesselheim, 2017; Duhig et al., 2018; Ernst and Young, 2018b; Faulkner et al., 2018; Salzman et al., 2018; Goncalves et al., 2018; Drummond et al., 2019; Mahendraratnam et al., 2019; Moradi et al., 2019; Patel et al., 2019). Furthermore, this framework should entail a clear structure to initiate payments, specify the data collection process with attention to ownership of the data and foreseeing regular data audits, establish a defined management framework, define the funding arrangements of the agreement and clearly state the opportunities for appeal when requirements are not met (Adamski et al., 2010; McCabe et al., 2010; Williamson and Thomson, 2010; Klemp et al., 2011; Ferrario and Kanavos, 2013; Garrison Jr et al., 2013; Annemans and Pani, 2017; Clopes et al., 2017; EXPH, 2018; Wenzl and Chapman, 2019). Most importantly, the governance procedures should indicate the standardised decision-making criteria that are used to support the adjustment of payments based on the outcomes achieved (Coulton et al., 2012; Kornfeld et al., 2013; Gerkens et al., 2017; Kanavos et al., 2017; Makady et al., 2019).

This structure could be reinforced by a core steering committee to establish and oversee the agreement as recommended by the ISPOR good practices for performance-based risk-sharing arrangements task force (Chapman et al., 2003; Garrison Jr et al., 2013). The members of this committee could be the payer, the health technology assessment agency, the manufacturer and the healthcare provider as they are deemed crucial due to their budgetary and/or administrative responsibilities within these agreements (Menon et al., 2011; Ferrario and Kanavos, 2013; Garrison Jr et al., 2013; Drummond, 2015). Additionally, this committee could provide minimum transparency, without disclosing sensitive financial information, on the process of OBA establishment, the governance framework, the progress of data collection and the gathered real-world evidence by annual reports to ensure that all stakeholders groups are held accountable (Hutton et al., 2007; Boggild et al., 2009; McCabe et al., 2010; Raftery, 2010; Cole et al., 2019; Wenzl am]nd Chapman, 2019). Furthermore, studies investigating both experiences with OBAs and stakeholder perspectives highlight the possibility of including an external advisory board with independent experts, having no affiliation toward the payer or manufacturer, such as researchers, statisticians, health economists, IT experts, healthcare professionals and patient representatives to evaluate proposed schemes (while respecting the necessary confidentiality) on their implementation, the data collection process and the analysis of the gathered evidence (Hutton et al., 2007; Adamski et al., 2010; Menon et al., 2011; Thompson et al., 2016; Gerkens et al., 2017; Alliance for Regenerative Medicine, 2019; Makady et al., 2019; Wenzl and Chapman, 2019). The enablement of such a governance structure will require a multi-stakeholder approach guaranteeing good communication and clear entry points into the relevant OBA processes for every stakeholder (Adamski et al., 2010; Klemp et al., 2011; Menon et al., 2011; Thompson et al., 2016; AMCP, 2019; Macaulay and Turkstra, 2019).

However, collaboration between different stakeholders may be complicated by the underlying interests of every stakeholder group and their respective incentives ( de Pouvourville, 2006; Carlson et al., 2009; Puig-Peiró et al., 2011; Neumann et al., 2011; Brennan and Wilson, 2014; Barlas, 2016a; Carr and Bradshaw, 2016; Kiernan, 2016; Proach et al., 2016; Van De Vijver et al., 2016; NEHI, 2017; Value in Health, 2017; Cole et al., 2019; FoCUS, 2019a; Federici et al., 2019; Pace et al., 2019; Kannarkat et al., 2020). Therefore, several authors recommend performing an analysis of the interests of all stakeholders at all stages of the agreement allowing the declaration of possible conflicts of interests and affiliations before the start of the agreement (Chapman et al., 2003; Raftery, 2010; Menon et al., 2011; Clopes et al., 2017; Gerkens et al., 2017; Makady et al., 2019). Although payers, developers and providers have several positive incentives to engage in OBAs, different negative incentives and conflicting interests are reported in literature and may be an additional barrier to OBA implementation. However, several facilitators are proposed to mitigate these negative incentives (Table 2). Next to diverging interests and incentives, a survey with payers and manufacturers showed that a barrier for the collaboration between stakeholders during OBAs is the lack of trust between healthcare professionals, payers and manufacturers (Mahendraratnam et al., 2019). This trust is imperative to ensure that all stakeholders trust that the data was gathered and analyzed in an unbiased manner and will be shared correctly (Garrison Jr et al., 2015; AMCP, 2017). Therefore, stakeholders are encouraged to act in a trustworthy manner by being transparent about their goals and respect the terms dictated in the agreement (Thompson et al., 2016; Mahendraratnam et al., 2019). To enhance trust, several authors propose working with an independent third party such as academic institutions or non-profit, publicly funded organisations to perform all stages of the data collection process (Adamski et al., 2010; McCabe et al., 2010; Stafinski et al., 2010; Menon et al., 2011; Goldenberg and Bachman, 2012; Ferrario and Kanavos, 2013; Drummond, 2015; Thompson et al., 2016; Kanavos et al., 2017; Bouvy et al., 2018; EXPH, 2018; Mahendraratnam et al., 2019; Wenzl and Chapman, 2019). The importance of such independence was shown by the decision made by the scientific advisory group, consisting of representatives with high interest in continued access to the medicines, of the United Kingdom multiple sclerosis (MS) agreement to maintain reimbursement even though many patients experienced worse outcomes (Raftery, 2010; Towse et al., 2012; Gibson and Lemmens, 2014).

**TABLE 2:** Conflicting interests and incentives of stakeholders during outcome-based agreements and possible facilitators to mitigate negative incentives as reported in literature.

Stakeholder	Stakeholder interest	Incentive	Possible measures
Payer	Minimize impact on the healthcare budget Menon et al. (2010)	No incentive for robust data collection due to risk of price increase if better performance Towse and Garrison Jr (2010)	—
		No incentive for robust data collection once financial deal is done Bouvy et al. (2018)	
		Selection of high-risk patients who have higher treatment failure which leads to a higher financial return EXPH (2018), Kannarkat et al. (2020)	
Manufacturer	Maximize duration of market access and the number of patients treated Menon et al. (2010)	No incentive for robust data collection due to risk of price decrease if lower performance Towse and Garrison Jr (2010), Garrison Jr et al. (2013), Gerkens et al. (2017), Antonanzas et al. (2018), Makady et al. (2019), Wenzl and Chapman (2019)	Include incentives for data collection by rewarding better performance with payment increase Annemans and Pani (2017), Hettle et al. (2017), FoCUS (2019a)
		No incentive for robust data collection since competitors might take advantage (free-riding) Towse and Garrison Jr (2010), Ferrario and Kanavos (2013), Gerkens et al. (2017), Kanavos et al. (2017), Wenzl and Chapman (2019)	Make data collection a formal requirement. If not performed, convention is terminated Gerkens et al. (2017), Wenzl and Chapman (2019)
		Selection of low-risk patients who have lower treatment failure which leads to a higher financial return Kannarkat et al. (2020)	—
		Incentive to set higher initial price to compensate for higher uncertainty on return on investment due to possible price decreases Hutton et al. (2007), Adamski et al. (2010), Trueman et al. (2010), Carlson et al. (2011), Ferrario and Kanavos (2013), Garrison Jr et al. (2013), Gerkens et al. (2017), Kanavos et al. (2017), Wenzl and Chapman (2019)	—
Healthcare professional	Access to maximum number of treatment options Menon et al. (2010)	No incentive for robust data collection since it is not clear what the benefit of extra data for the clinic will be Pauwels et al. (2017), Maes et al. (2019)	Give healthcare professionals appropriate fees to cover data collection expenses Neumann et al. (2011), van de Wetering et al. (2017), FoCUS (2019b)
		No incentive for robust data collection due to risk of losing patients if treatment is not cost-effective Makady et al. (2019)	Make date collection a requirement to allow patient reimbursement Maes et al. (2019)
—	—	—	Increasingly engage physicians during the design of the agreement Menon et al. (2011), Annemans and Pani (2017)
Patient	Access to maximum number of treatment options Menon et al. (2010)	No incentive for robust data collection since cured patients will not be motivated to attend follow-up consultations Coulton et al. (2012), Touchot and Flume (2015), AMCP (2019), Maes et al. (2019)	Make co-pays dependent on patients attending follow-up appointments Coulton et al. (2012)

Implementation of OBAs with spread payments will be further complicated by the current experienced cost of implementing and managing OBAs with upfront payment. The cost of managing registries for OBA-related data collection has been estimated to be around one million euros in Italy (Garattini et al., 2015). A similar cost estimate was made for the reimbursement of CAR-T cell therapy in the United Kingdom with a MEA, compared with CAR-T cell therapy without a MEA, which would result in an incremental administrative burden of £871.707 over 10 years (Kefalas et al., 2018). However, these costs can be highly variable since the MS risk-sharing scheme in the United Kingdom was estimated to cost nearly £50 million annually (Sudlow and Counsell, 2003; Raftery, 2010) while the costs of the OBA for gefitinib in Catalonia were estimated to be negligible due to the readiness of the organisational structures (Clopes et al., 2017). The majority of costs are attributed to the increase in personnel time and required personnel to perform data collection, the cost of the infrastructure required for data collection, guaranteeing compliance with the established agreement and initiating payments (Sudlow and Counsell, 2003; Raftery, 2010; Loveman et al., 2011; Garrison Jr et al., 2013; Garattini et al., 2015; Garrison Jr et al., 2015; Clopes et al., 2017; Jorgensen and Kefalas, 2017; Macaulay and Hettle, 2017; Kefalas et al., 2018; Mailankody and Bach, 2018; FoCUS, 2019a; Lorente et al., 2019; Mundy et al., 2019). This burden is predominantly experienced by the payer and the healthcare professionals who are responsible for overseeing the agreement and performing data collection. Payers struggle with their administrative capacity to handle (multiple) agreements under current staffing levels (Adamski et al., 2010; FoCUS, 2019b; Jorgensen et al., 2019; van de Wetering et al., 2017). Healthcare professionals, specifically clinicians, nurses, pharmacists and more broadly the hospital administration and finance department, are increasingly burdened with administrative tasks to collect data (Williamson and Thomson, 2010; Franken et al., 2014; Gibson and Lemmens, 2014; Garattini et al., 2015; Kiernan, 2016; Nazareth et al., 2017; Brown et al., 2018; Carr et al., 2018; Schaffer et al., 2018; Antonanzas et al., 2019). They require an increase in staff resources by hiring dedicated healthcare staff or increasing personnel time for management of agreements (Coulton et al., 2012; Goodman et al., 2019; Macaulay and Turkstra, 2019). This was evident from the United Kingdom MS agreement which required hiring approximately 150 additional nurses and an increase in neurology consultations (Sudlow and Counsell, 2003; Garattini and Casadei, 2011). Moreover, Jørgensen et al. emphasize that staff resource shortages are more apparent in primary care which complicates the data collection in the ideal situation where patients are cured after one-time treatment (Jorgensen et al., 2019).

Efficient governance can partly alleviate the administrative burden and cost of OBAs. Therefore, studies investigating the current experience with OBAs and their respective administrative burden emphasize the creation of a streamlined governance process similar for every agreement as being crucial to reduce the cumulative burden of all agreements (Espin et al., 2011; Ferrario et al., 2011; Garrison Jr et al., 2013; Faulkner et al., 2016; Macaulay and Turkstra, 2019; Towse and Fenwick, 2019). Furthermore, lessons learned from the administrative burden experienced in the Belgian healthcare system indicates that utilisation and optimisation of existing administrative systems is essential to ensure a streamlined implementation of OBAs with a reduced burden for healthcare staff (Gerkens et al., 2017). This will require an initial investment in an adequate IT infrastructure and continued investment in the education and support of experienced staff in data collection and OBA management (Menon et al., 2010; Franken et al., 2014; Garrison Jr et al., 2015; Hettle et al., 2017; Schaffer et al., 2018; Antonanzas et al., 2019; Federici et al., 2019; Makady et al., 2019). Adamski et al. stress that efficient governance also includes clear agreements on how the OBAs should be funded (Adamski et al., 2010). The costs for upgrading the data collection infrastructure, the cost of personnel training, the cost of additional personnel (time) and the general management of the scheme could be included in the underlying agreement as transaction costs to the healthcare system (Chapman et al., 2003; Coulton et al., 2012). The manufacturer has been proposed as funder due to the incapacity of government payers to fund the additional efforts to establish MEAs (Edlin et al., 2014; Thompson et al., 2016). Others suggest the payer as responsible for the financing of agreements since they are the ultimate buyers of the technologies that will benefit the entire healthcare system (McCabe et al., 2010; Stafinski et al., 2010; Menon et al., 2011; Edlin et al., 2014; Thompson et al., 2016). Several authors discuss shared responsibility for funding the agreement by both the payer and the manufacturer and possibly research organisations or granting agencies (McCabe et al., 2010; Stafinski et al., 2010; Thompson et al., 2016). Irrespective of the funding party, the governance structure must include who will be responsible for the funding of every task required to enable OBAs (Touchot and Flume, 2015; Carlson et al., 2017; Hettle et al., 2017; Schaffer et al., 2018; Value in Health, 2017).

## Discussion

By including literature on both implementation of spread payments as on implementation of traditional OBAs with upfront payments, we were able to provide a full overview of all barriers that outcome-based spread payments will have to overcome to be successfully implemented. Although outcome-based spread payments can be a promising mechanism for market access of high-cost, one-shot therapies, several challenges will need to be resolved to enable their use across Europe. First, a value of information analysis should be systematically performed to confirm that remaining uncertainties may be solved by engaging in an OBA (Towse and Garrison Jr, 2010; Gerkens et al., 2017). If the value of information analysis encourages the use of an OBA to mitigate uncertainties and the developer and payer choose to spread payments over time to mitigate unaffordability, both parties will have to agree on an adequate structure for spreading payments corrected by outcomes. Agreement on payment structure will need to be reached on the payment amount per installment, the time frame during which payments will be made and how payments should be adapted based on achieved real-world performance (Hettle et al., 2017; Yeung et al., 2017; Faulkner et al., 2018; Barlow et al., 2019).

This agreement is complicated by the choice for either individual-level or population-based outcome correction which depends on the existing data collection infrastructure and if the goal of the agreement is to perform real-time adjustment of individual payments or to adjust payments based on real-world effectiveness in the complete population (Williamson and Thomson, 2010; Kleinke and McGee, 2015). The data collection infrastructure will both determine the additional burden for healthcare providers and payers of using outcome-based spread payments, and the decisions on optimal study design and outcome selection. Therefore, an upgrade of the current infrastructure and cross-country collaboration may be required to decrease collection burden, allow high-quality data collection and may support data sharing (Ferrario and Kanavos, 2013; Faulkner et al., 2016; Alliance for Regenerative Medicine, 2019).

On an individual level, the payment could be adapted or stopped if the expected outcome is not reached (Edlin et al., 2014; Jorgensen and Kefalas, 2017; FoCUS, 2019a). Outcome correction of spread payments on population-level can be proportional to the difference between real-world and clinical trial performance as measured with a core set of (validated surrogate) outcomes used in clinical trials (Garrison Jr et al., 2013; Yeung et al., 2017; Cole et al., 2019; Maes et al., 2019). While adjusting installment payments on an individual-level can occur in real-time, population-based payment adjustment can only occur once per payment period but may inform product performance in the whole population (Launois et al., 2014; Wenzl and Chapman, 2019) and may better comply with GDPR regulations since only aggregated patient data will need to be shared (Menner and Lewandowska, 2018; Alliance for Regenerative Medicine, 2019). The duration of spread payments should be limited in time in order not to burden future generations and be edged on the expected and measured duration of benefit (Edlin et al., 2014; Maes et al., 2019). Furthermore, horizon scanning, identifying novel products entering the market, will be required to ensure that agreements do not inhibit future market competition (Menon et al., 2011; Pauwels et al., 2017; Hanna et al., 2018; Wenzl and Chapman, 2019). Schaffer et al. propose the use of clauses that re-open the contract after exclusivity loss or entry of a competitive product allowing an adjustment of the payment amount or complete stop of the contract to adapt to an evolving competitive environment (Schaffer et al., 2018).

Implementation of outcome-based spread payments is further impeded by the required additional tracking of payments over time between healthcare provider, payer and developer. However, this may stimulate finetuning of current financial flows and may even trigger a change of purchasing party to payer instead of healthcare provider (Gerkens et al., 2017; Schaffer et al., 2018; Wenzl and Chapman, 2019). Furthermore, payer accounting systems may need to be changed to solve challenges with recording payments over multiple years considering current 12-months budget cycles, and European and national accounting rules (Alliance for Regenerative Medicine, 2019; Maes et al., 2019). Additionally, the negative influence of spreading payments on revenue streams and financial obligations of the developer currently limits the use of outcome-based spread payments which may be especially important for small and medium enterprises with less financial reserve (Edlin et al., 2014; Hettle et al., 2017; Kanavos et al., 2017).

**FIGURE 2:**
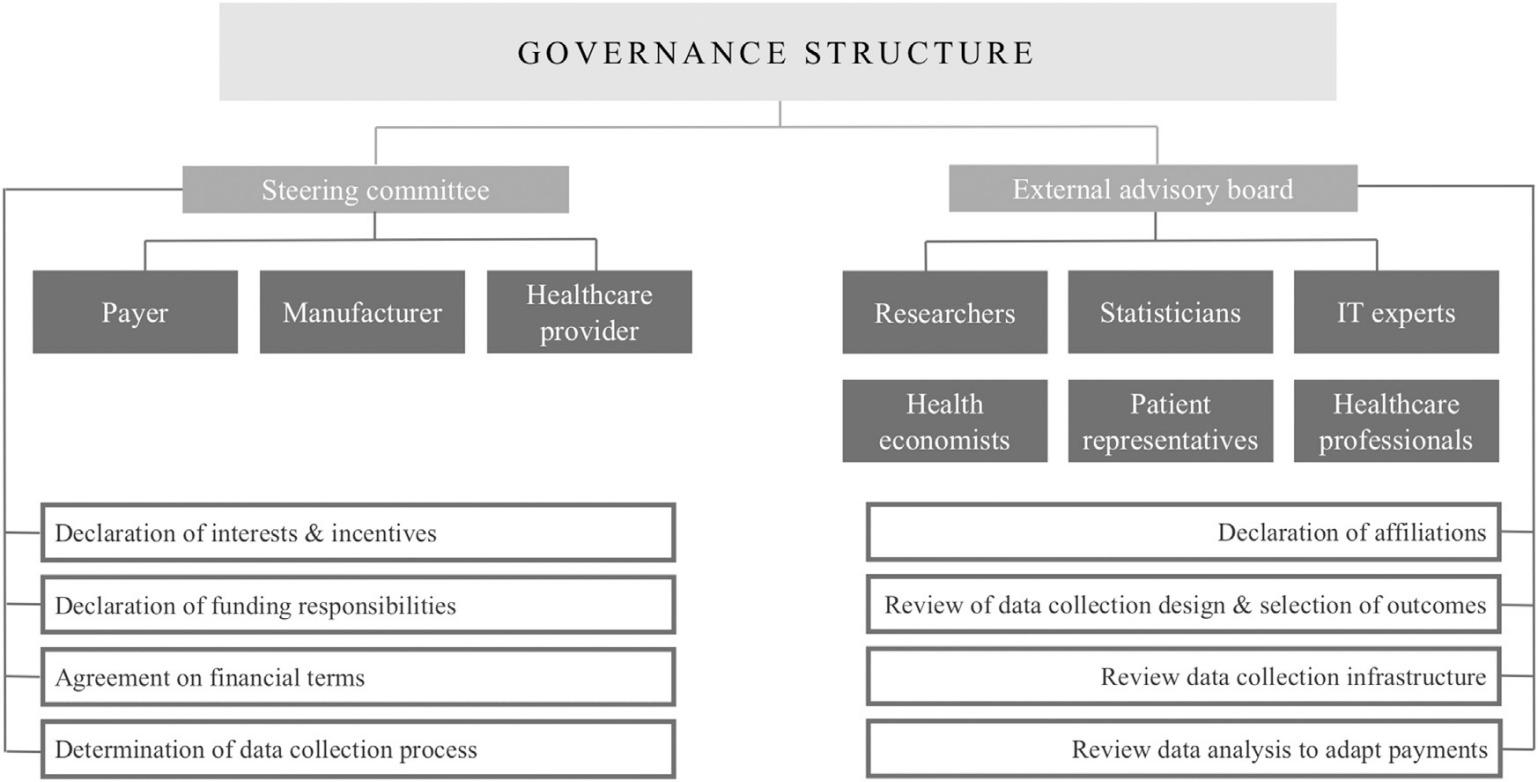
Proposed governance structure for the implementation of outcome-based spread payments.

To enable the complete system of outcome-based spread payments, a steering committee and external advisory board could be developed as a general, systematic governance structure paying attention to stakeholders’ roles and responsibilities as proposed in Figure 2. This governance structure will require the declaration of interests and incentives of all stakeholders, as shown in Table 1, and should detail the responsibilities for funding of the agreement which may be the manufacturer, the payer or a combination of both (Hutton et al., 2007; Adamski et al., 2010; Menon et al., 2011; Garrison Jr et al., 2013; Thompson et al., 2016; Jorgensen and Kefalas, 2017; Makady et al., 2019).

This systematic literature review provides an overview of barriers and opportunities for implementation of outcome-based spread payments. However, existing literature specifically discussing outcome-based spread payments was limited. Therefore, we included reported barriers on outcome correction, possibly of lower relevance, from publications discussing traditional OBAs with upfront payments to ensure a complete overview. Furthermore, barriers and opportunities discussed were included from both empirical research and from authors’ and stakeholders’ perspectives as the aim of this study was to guarantee all possible barriers were identified. The independent review of titles and abstracts by two researchers reduced subjectivity; however, full text review and data extraction was performed by one researcher (SM) which did not allow additional cross-checking of findings. Furthermore, diverse terminology employed for MEAs, OBAs and spread payments may have led to loss of relevant sources and publication bias may have been introduced due to the confidential nature of MEAs. Other relevant records could have been missed since only English literature was included and relevant gray literature, identified with hand-searching, was overlooked. Furthermore, the article focused on barriers experienced by European single-payer high-income countries and does not include challenges encountered by other non-European jurisdictions and lower income countries. Although several solutions to the identified barriers have been proposed in literature, implementation of outcome-based spread payments still faces several hurdles and proposed opportunities in literature have not yet been tested for their applicability in practice. Therefore, future research is needed to develop recommendations for the implementation of this novel reimbursement structure by investigating how remaining barriers can be overcome and how proposed opportunities may be implemented in practice.

In conclusion, outcome-based spread payments may be a promising solution to allow affordable access of high-cost, one-shot possibly curative therapies. However, their implementation in Europe will require several organizational changes to overcome the challenges payers, manufacturers and healthcare providers face. These challenges are mainly caused by the struggle to reach multi-stakeholder agreement on financial terms to spread payments, implementing an adequate governance framework, setting up data collection and existing legislative obstructions. However, spread payments adjusted by outcome data collected within automated registries and overseen by a governance committee and external advisory board may alleviate several barriers and may support the reimbursement of highly innovative therapies.

## Author Contributions

SM, WV, SS and IH were involved in the development of the study design. SM and SN performed the title and abstract screening. MS performed the full text review and analysis of included records. SM, WV, SS and IH participated in meetings and reviewed materials. SM produced the first draft of the manuscript, which was subsequently revised and finalized with all authors. All authors approved the final manuscript.

## Funding

This work was supported by a PhD fellowship strategic basic research of the Research Foundation – Flanders (1S70720N).

## Conflict of Interest

SS has provided advice to Novartis about the design of a managed entry agreement for an advanced therapy medicinal product. VW has led a Pfizer-sponsored Belgian national Round Table gathering business and societal perspectives on high-priced gene therapies.

The remaining authors declare that the research was conducted in the absence of any commercial or financial relationships that could be construed as a potential conflict of interest.
