# Supplementary material for: Barriers and Opportunities for Implementation of Outcome-Based Spread Payments for High-Cost, One-Shot Curative Therapies
Source: Front Pharmacol. 2020 Dec 8;11:594446. doi: 10.3389/fphar.2020.594446 (PMC7753155; doi:10.3389/fphar.2020.594446)
Supplement: Supplementary file 1 [file table1.docx]

Supplementary Material

# Supplementary Table 1 – Search query

A AND (B OR C OR D)

| **A: Managed entry agreement** | | |
| --- | --- | --- |
| Concept | MeSH/Emtree | Free text (search in title and abstract) |
| Managed entry | / | ‘managed entry’ OR ‘managed access’ OR ‘patient access’ OR ‘risk sharing’ OR ‘risk-sharing’ OR ‘value-based’ |
| Reimbursement | / | ‘reimbursement’ OR ‘sustainable financing’ OR ‘debt financing’ |
| **B: Outcome-based agreement** | | |
| Concept | MeSH/Emtree | Free text (search in title and abstract) |
| Performance based | / | ‘performance based’ OR ‘performance-based’ OR ‘performance linked’ OR ‘performance-linked’ OR ‘pay for performance’ OR ‘pay-for-performance’ OR ‘paying for performance’ OR ‘paying-for-performance’ OR ‘payment model’ OR ‘payment models’ |
| Outcome based | / | ‘outcome based’ OR ‘outcome-based’ OR outcomes based OR outcomes-based OR ‘coverage with evidence’ OR ‘access with evidence’ |
| **C: Annuity-based payment** | | |
| Concept | MeSH/Emtree | Free text (search in title and abstract) |
| Annuity | / | ‘annuity’ OR ‘annuities’ OR ‘leasing’ OR ‘lease’ OR ‘amortization’ OR ‘renting’ OR ‘rent’ |
| **D: Transversal budget** | | |
| Concept | MeSH/Emtree | Free text (search in title and abstract) |
| Transversal | / | ‘transversal budget’ OR ‘transversal budgets’ OR ‘transversal budgeting’ OR ‘pooled budget’ OR ‘pooled budgets’ OR ‘pooled budgeting’ OR ‘combined budget’ OR ‘combined budgets’ OR ‘combined budgeting’ OR ‘cross-silo budget’ OR ‘cross-silo budgets’ OR ‘cross-silo budgeting’ OR ‘silo budget’ OR ‘silo budgets’ OR ‘silo budgeting’ |

**Complete search string:**

*PubMed database:*

("performance based"[Title/Abstract] OR "performance-based"[Title/Abstract] OR "performance linked"[Title/Abstract] OR "performance-linked"[Title/Abstract] OR "pay for performance"[Title/Abstract] OR "pay-for-performance"[Title/Abstract] OR "paying for performance"[Title/Abstract] OR "paying-for-performance"[Title/Abstract] OR "outcome based"[Title/Abstract] OR "outcome-based"[Title/Abstract] OR "outcomes based"[Title/Abstract] OR "outcomes-based"[Title/Abstract] OR "coverage with evidence"[Title/Abstract] OR "access with evidence"[Title/Abstract] OR "payment model"[Title/Abstract] OR "payment models"[Title/Abstract] OR "annuity"[Title/Abstract] OR "annuities"[Title/Abstract] OR "leasing"[Title/Abstract] OR "lease"[Title/Abstract] OR "rent"[Title/Abstract] OR "renting"[Title/Abstract] OR "amortization"[Title/Abstract] OR "pooled budget"[Title/Abstract] OR "pooled budgets"[Title/Abstract] OR "pooled budgeting"[Title/Abstract] OR "combined budget"[Title/Abstract] OR "combined budgets"[Title/Abstract] OR "combined budgeting"[Title/Abstract] OR "cross-silo budget"[Title/Abstract] OR "cross-silo budgets"[Title/Abstract] OR "cross-silo budgeting"[Title/Abstract] OR "silo budget"[Title/Abstract] OR "silo budgets"[Title/Abstract] OR "silo budgeting"[Title/Abstract]) AND ("reimbursement"[Title/Abstract] OR "sustainable financing"[Title/Abstract] OR "debt financing"[Title/Abstract] OR "patient access"[Title/Abstract] OR "managed entry"[Title/Abstract] OR "managed access"[Title/Abstract] OR "risk sharing"[Title/Abstract] OR "risk-sharing"[Title/Abstract] OR "value based"[Title/Abstract] OR "value-based"[Title/Abstract])

*Embase database:*

('performance based':ab,ti OR 'performance-based':ab,ti OR 'performance linked':ab,ti OR 'performance-linked':ab,ti OR 'pay for performance':ab,ti OR 'pay-for-performance':ab,ti OR 'paying for performance':ab,ti OR 'paying-for-performance':ab,ti OR 'payment model':ab,ti OR 'payment models':ab,ti OR 'outcome based':ab,ti OR 'outcome-based':ab,ti OR 'coverage with evidence':ab,ti OR 'access with evidence':ab,ti OR 'annuity':ab,ti OR 'annuities':ab,ti OR 'leasing':ab,ti OR 'lease':ab,ti OR 'renting':ab,ti OR 'rent':ab,ti OR 'amortization':ab,ti OR 'transversal budget':ab,ti OR 'transversal budgets':ab,ti OR 'transversal budgeting':ab,ti OR 'pooled budget':ab,ti OR 'pooled budgets':ab,ti OR 'pooled budgeting':ab,ti OR 'combined budget':ab,ti OR 'combined budgets':ab,ti OR 'combined budgeting':ab,ti OR 'cross-silo budget':ab,ti OR 'cross-silo budgets':ab,ti OR 'cross-silo budgeting':ab,ti OR 'silo budget':ab,ti OR 'silo budgets':ab,ti OR 'silo budgeting':ab,ti) AND ('managed entry':ab,ti OR 'managed access':ab,ti OR 'patient access':ab,ti OR 'risk sharing':ab,ti OR 'risk-sharing':ab,ti OR 'reimbursement':ab,ti OR 'sustainable financing':ab,ti OR 'debt financing':ab,ti OR 'value-based':ab,ti OR 'value based':ab,ti)
