# Supplementary material for: Barriers and Opportunities for Implementation of Outcome-Based Spread Payments for High-Cost, One-Shot Curative Therapies
Source: Front Pharmacol. 2020 Dec 8;11:594446. doi: 10.3389/fphar.2020.594446 (PMC7753155; doi:10.3389/fphar.2020.594446)
Supplement: Supplementary file 2 [file table2.docx]

Supplementary Material

# Supplementary Table 2 - Overview of publications discussing outcome-based agreements and/or (outcome-based) spread payments

| **First author** | **Year** | **Source** | **Agreement type for which barriers & potential opportunities for implementation were discussed** | **Categories for which barriers and potential opportunities were discussed** |
| --- | --- | --- | --- | --- |
| Kannarkat et al. | 2020 | (1) | Outcome-based agreements | Governance, organisation of data collection, outcome correction |
| Moradi et al. | 2019 | (2) | Spread payments Outcome-based agreements | Governance |
| Jönsson et al. | 2019 | (3) | Outcome-based spread payments Outcome-based agreements | Governance, process of data collection, organisation of data collection, payment structure, outcome correction |
| AMCP Partnership Forum | 2019 | (4) | Outcome-based spread payments Outcome-based agreements | Governance, process of data collection, organisation of payments, payment structure |
| Barlow et al. | 2019 | (5) | Spread payments Outcome-based spread payments Outcome-based agreements | Governance, organisation of payments |
| Makady et al | 2019 | (6) | Outcome-based agreements | Governance, process of data collection, organisation of data collection |
| Jorgensen et al. | 2019 | (7) | Outcome-based spread payments Outcome-based agreements | Governance, organisation of data collection |
| Lorente et al. | 2019 | (8) | Outcome-based agreements | Governance, process of data collection, organisation of data collection, organisation of payments |
| Makady et al. | 2019 | (9) | Outcome-based agreements | Governance, process of data collection, outcome correction |
| Mundy et al. | 2019 | (10) | Outcome-based agreements | Governance, process of data collection, organisation of data collection |
| Patel et al. | 2019 | (11) | Outcome-based spread payments Outcome-based agreements | Governance |
| Infante et al. | 2019 | (12) | Spread payments Outcome-based agreements | Payment structure, organisation of payments, outcome correction |
| Pace et al. | 2019 | (13) | Outcome-based agreements | Governance, process of data collection, organisation of payments |
| Macaulay et al. | 2019 | (14) | Outcome-based agreements | Governance, organisation of data collection |
| Federici et al. | 2019 | (15) | Outcome-based agreements | Governance, process of data collection, organisation of payments, outcome correction |
| Goodman et al. | 2019 | (16) | Outcome-based agreements | Governance, process of data collection, organisation of data collection, organisation of payments |
| Antonanzas et al. | 2019 | (17) | Outcome-based agreements | Governance, organisation of data collection |
| Towse et al. | 2019 | (18) | Spread payments Outcome-based spread payments Outcome-based agreements | Governance, process of data collection, payment structure, organisation of payments, outcome correction |
| Jorgensen et al. | 2019 | (19) | Outcome-based spread payments  Outcome-based agreements | Governance, process, of data collection organisation of data collection, organisation of payments |
| Mahendraratnam et al. | 2019 | (20) | Outcome-based agreements | Governance, process of data collection, organisation of data collection, organisation of payments, outcome correction |
| Drummond et al. | 2019 | (21) | Outcome-based spread payments Outcome-based agreements | Governance, payment structure, organisation of payments, outcome correction |
| Maes et al. | 2019 | (22) | Spread payments Outcome-based spread payments Outcome-based agreements | Governance, process of data collection, organisation of data collection, payment structure, organisation of payments, outcome correction |
| Cole et al. | 2019 | (23) | Outcome-based agreements | Governance, process of data collection, organisation of data collection, payment structure, organisation of payments, outcome correction |
| Alliance for Regenerative Medicine | 2019 | (24) | Spread payments Outcome-based spread payments Outcome-based agreements | Governance, process of data collection, organisation of data collection, organisation of payments, outcome correction |
| MIT’s NEW Drug Development ParadIGmS (NEWDIGS) program | 2019 | (25) | Spread payments Outcome-based spread payments Outcome-based agreements | Governance, process of data collection, organisation of data collection, payment structure, organisation of payments, outcome correction |
| Wenzl et al. | 2019 | (26) | Outcome-based agreements | Governance, process of data collection, organisation of data collection, organisation of payments, outcome correction |
| Kim et al. | 2018 | (27) | Outcome-based agreements | Governance, process of data collection, organisation of data collection |
| Salzman et al. | 2018 | (28) | Spread payments Outcome-based agreements | Governance, payment structure, organisation of payments |
| Pham et al. | 2018 | (29) | Outcome-based agreements | Process of data collection |
| Robinson et al. | 2018 | (30) | Outcome-based agreements | Governance, process of data collection, organisation of data collection |
| Oren et al. | 2018 | (31) | Outcome-based agreements | Organisation of payments, outcome correction |
| Schmetz et al. | 2018 | (32) | Outcome-based agreements | Process of data collection, organisation of data collection |
| Kefalas et al. | 2018 | (33) | Outcome-based spread payments Outcome-based agreements | Governance, organisation of data collection |
| Hanna et al. | 2018 | (34) | Spread payments Outcome-based spread payments Outcome-based agreements | Governance, process of data collection, organisation of data collection, payment structure, organisation of payments, outcome correction |
| Nevins et al. | 2018 | (35) | Outcome-based spread payments | Organisation of payments |
| Sachs et al. | 2018 | (36) | Spread payments Outcome-based agreements | Organisation of data collection, payment structure, organisation of payments, outcome correction |
| Maskineh et al. | 2018 | (37) | Outcome-based agreements | Governance, organisation of data collection, outcome correction |
| Bouvy et al. | 2018 | (38) | Outcome-based agreements | Governance, process of data collection, organisation of data collection, organisation of payments, outcome correction |
| Lidonnici et al. | 2018 | (39) | Outcome-based agreements | Governance |
| Richardson et al. | 2018 | (40) | Spread payments | Organisation of payments |
| Brown et al. | 2018 | (41) | Outcome-based agreements | Governance, outcome correction |
| Sandhu et al. | 2018 | (42) | Outcome-based agreements | Process of data collection |
| Antonanzas et al. | 2018 | (43) | Outcome-based agreements | Outcome correction |
| Goncalves et al. | 2018 | (44) | Outcome-based agreements | Governance, process of data collection, organisation of data collection |
| Goldenberg et al. | 2018 | (45) | Outcome-based agreements | Organisation of data collection |
| Duhig et al. | 2018 | (46) | Outcome-based agreements | Process of data collection, organisation of data collection, organisation of payments |
| Urbinati et al. | 2018 | (47) | Outcome-based agreements | Organisation of data collection |
| Carr et al. | 2018 | (48) | Spread payments  Outcome-based spread payments  Outcome-based agreements | Governance, organisation of data collection, payment structure, outcome correction |
| Jorgensen et al. | 2018 | (49) | Outcome-based spread payments | Process of data collection |
| Danzon et al. | 2018 | (50) | Spread payments Outcome-based spread payments | Governance, process of data collection, outcome structure, outcome correction |
| Faulkner et al. | 2018 | (51) | Spread payments Outcome-based spread payments | Payment structure, organisation of payments, outcome correction |
| Senior et al. | 2018 | (52) | Spread payments Outcome-based spread payments Outcome-based agreements | Payment structure, organisation of payments, outcome correction |
| Menner et al. | 2018 | (53) | Outcome-based agreements | Organisation of data collection |
| Stirnadel-Farrant et al. | 2018 | (54) | Outcome-based agreements | Governance, process of data collection, organisation of data collection |
| Tuffaha et al. | 2018 | (55) | Spread payments Outcome-based spread payments Outcome-based agreements | Governance, process of data collection, payment structure, organisation of payments, outcome correction |
| Mailankody et al. | 2018 | (56) | Outcome-based agreements | Governance, process of data collection, outcome correction |
| European Commission | 2018 | (57) | Outcome-based agreements | Governance, process of data collection, organisation of data collection, outcome correction |
| Ernst & Young | 2018 | (58) | Outcome-based agreements | Governance, process of data collection, organisation of data collection |
| Ernst & Young | 2018 | (59) | Outcome-based agreements | Governance, process of data collection, organisation of data collection |
| MIT’s NEW Drug Development ParadIGmS (NEWDIGS) program | 2018 | (60) | Outcome-based spread payments Outcome-based agreements | Governance, process of data collection, organisation of data collection, payment structure, organisation of payments, outcome correction |
| AMCP Partnership Forum | 2017 | (61) | Outcome-based agreements | Governance, process of data collection, organisation of data collection |
| Jorgensen et al. | 2017 | (62) | Spread payments Outcome-based spread payments | Governance, organisation of data collection, payment structure, organisation of payments, outcome correction |
| Holleman et al. | 2017 | (63) | Outcome-based agreements | Outcome correction |
| Calabrese et al. | 2017 | (64) | Outcome-based agreements | Governance |
| Macaulay et al. | 2017 | (65) | Spread payments Outcome-based spread payments Outcome-based agreements | Governance |
| Pauwels et al. | 2017 | (66) | Outcome-based agreements | Governance, process of data collection, organisation of data collection, organisation of payments, outcome correction |
| Nazareth et al. | 2017 | (67) | Outcome-based agreements | Governance, process of data collection, organisation of data collection |
| Spark et al. | 2017 | (68) | Spread payments Outcome-based spread payments Outcome-based agreements | Organisation of payments |
| Yu et al. | 2017 | (69) | Outcome-based agreements | Process of data collection, organisation of data collection |
| Carlson et al. | 2017 | (70) | Spread payments Outcome-based spread payments Outcome-based agreements | Governance, process of data collection, payment structure, organisation of payments, outcome correction |
| Goble et al. | 2017 | (71) | Outcome-based agreements | Governance, process of data collection, organisation of data collection, outcome correction |
| Hettle et al. | 2017 | (72) | Spread payments Outcome-based spread payments Outcome-based agreements | Governance, process of data collection, payment structure, organisation of payments, outcome correction |
| Van de Wetering et al. | 2017 | (73) | Outcome-based agreements | Governance, organisation of data collection |
| Toumi et al. | 2017 | (74) | Outcome-based agreements | Process of data collection, organisation of data collection, organisation of payments, outcome correction |
| Yeung et al. | 2017 | (75) | Outcome-based agreements | Process of data collection, organisation of data collection |
| Yeung et al. | 2017 | (76) | Spread payments Outcome-based spread payments | Organisation of data collection, payment structure, organisation of payments, outcome correction |
| Clopes et al. | 2017 | (77) | Outcome-based agreements | Governance, process of data collection, organisation of data collection, outcome correction |
| Hampson et al. | 2017 | (78) | Spread payments Outcome-based spread payments Outcome-based agreements | Governance, process of data collection, payment structure, organisation of payments, outcome correction |
| Kanavos et al. | 2017 | (79) | Outcome-based agreements | Governance, process of data collection, organisation of data collection, organisation of payments |
| Schaffer et al. | 2017 | (80) | Spread payments Outcome-based spread payments | Process of data collection, organisation of data collection, payment structure, organisation of payments, outcome correction |
| Kazi et al. | 2017 | (81) | Outcome-based agreements | Process of data collection |
| Duke-Margolis Center for Health Policy Conference Center | 2017 | (82) | Outcome-based agreements | Governance, process of data collection, organisation of data collection, outcome correction |
| Annemans et al. | 2017 | (83) | Outcome-based agreements | Governance, process of data collection, organisation of data collection, outcome correction |
| Gerkens et al. | 2017 | (84) | Outcome-based agreements | Governance, process of data collection, organisation of data collection, organisation of payments, outcome correction |
| Slocomb et al. | 2017 | (85) | Spread payments Outcome-based spread payments Outcome-based agreements | Organisation of data collection, payment structure, organisation of payments, outcome correction |
| PricewaterhouseCoopers (PWC) | 2017 | (86) | Outcome-based agreements | Governance, process of data collection, organisation of data collection |
| Fox et al. | 2017 | (87) | Outcome-based agreements | Process of data collection, organisation of data collection, outcome correction |
| Seeley et al. | 2017 | (88) | Outcome-based agreements | Process of data collection, organisation of data collection, organisation of payments |
| Network For Excellence in Health Innovation | 2017 | (89) | Outcome-based agreements | Governance, process of data collection, organisation of data collection, outcome correction |
| Marsden et al. | 2017 | (90) | Spread payments Outcome-based spread payments | Process of data collection, organisation of data collection, payment structure, organisation of payments, outcome correction |
| Carroll et al. | 2016 | (91) | Outcome-based agreements | Governance |
| Proach et al. | 2016 | (92) | Spread payments | Organisation of data collection, organisation of payments |
| Carr et al. | 2016 | (93) | Spread payments Outcome-based spread payments | Governance, process of data collection, organisation of data collection, payment structure, outcome correction |
| Barlas et al. | 2016 | (94) | Outcome-based agreements | Governance, organisation of data collection |
| Pouwels et al. | 2016 | (95) | Outcome-based agreements | Process of data collection |
| Malik et al. | 2016 | (96) | Outcome-based agreements | Governance, process of data collection |
| Polimeni et al. | 2016 | (97) | Outcome-based agreements | Governance, outcome correction |
| Van De Vijver et al. | 2016 | (98) | Outcome-based agreements | Governance, organisation of data collection, outcome correction |
| Thompson et al. | 2016 | (99) | Outcome-based agreements | Governance, process of data collection, organisation of data collection, organisation of payments, outcome correction |
| Kiernan et al. | 2016 | (100) | Outcome-based agreements | Governance, process of data collection |
| Barlas et al. | 2016 | (101) | Outcome-based agreements | Governance |
| Montazerhodjat et al. | 2016 | (102) | Spread payments Outcome-based spread payments | Payment structure, organisation of payments, outcome correction |
| Abou-El-Enein et al. | 2016 | (103) | Spread payments Outcome-based spread payments | Process of data collection |
| Faulkner et al. | 2016 | (104) | Outcome-based agreements | Governance, process of data collection, organisation of data collection, organisation of payments, outcome correction |
| Pritchett et al. | 2015 | (105) | Outcome-based agreements | Process of data collection, organisation of data collection |
| De Rosa et al. | 2015 | (106) | Outcome-based agreements | Process of data collection, organisation of data collection |
| Lewis et al. | 2015 | (107) | Outcome-based agreements | Process of data collection, organisation of data collection |
| Navarria et al. | 2015 | (108) | Outcome-based agreements | Governance, process of data collection, organisation of data collection, organisation of payments, outcome correction |
| Mohseninejad et al. | 2015 | (109) | Outcome-based agreements | Governance, process of data collection, organisation of data collection |
| Lucas et al. | 2015 | (110) | Outcome-based agreements | Governance, organisation of data collection |
| Garrison et al. | 2015 | (111) | Outcome-based agreements | Governance, process of data collection, organisation of data collection, organisation of payments, outcome correction |
| Kocsis et al. | 2015 | (112) | Outcome-based agreements | Process of data collection |
| Drummond et al. | 2015 | (113) | Spread payments Outcome-based agreements | Governance, process of data collection, organisation of data collection payment structure, outcome correction |
| Kleinke et al. | 2015 | (114) | Spread payments Outcome-based spread payments | Process of data collection, organisation of data collection, payment structure, organisation of payments, outcome correction |
| Garattini et al. | 2015 | (115) | Outcome-based agreements | Governance, organisation of data collection, outcome correction |
| Montilla et al. | 2015 | (116) | Outcome-based agreements | Governance, process of data collection, organisation of data collection |
| Lu et al. | 2015 | (117) | Outcome-based agreements | Governance, organisation of data collection |
| Jorgensen et al. | 2015 | (118) | Outcome-based spread payments Outcome-based agreements | Organisation of data collection, payment structure, outcome correction |
| Touchot et al. | 2015 | (119) | Spread payments Outcome-based spread payments Outcome-based agreements | Governance, process of data collection, organisation of data collection, organisation of payments |
| Tuna et al. | 2014 | (120) | Outcome-based agreements | Organisation of data collection |
| Li et al. | 2014 | (121) | Outcome-based agreements | Governance, process of data collection, organisation of data collection |
| Carlson et al. | 2014 | (122) | Outcome-based agreements | Process of data collection, organisation of data collection, organisation of payments, outcome correction |
| Launois et al. | 2014 | (123) | Outcome-based agreements | Process of data collection, outcome correction |
| Vitry et al. | 2014 | (124) | Outcome-based agreements | Governance, organisation of data collection, organisation of payments |
| Gibson et al. | 2014 | (125) | Outcome-based agreements | Governance, organisation of data collection |
| Franken et al. | 2014 | (126) | Outcome-based agreements | Governance, process of data collection, organisation of data collection |
| Bibeau et al. | 2014 | (127) | Outcome-based agreements | Governance, organisation of data collection |
| Edlin et al. | 2014 | (128) | Spread payments Outcome-based spread payments | Governance, process of data collection, payment structure, outcome correction |
| Gottlieb et al. | 2014 | (129) | Spread payments Outcome-based spread payments | Governance, payment structure, organisation of payments, outcome correction |
| Basu et al. | 2014 | (130) | Spread payments | Payment structure, outcome correction |
| Brennan et al. | 2014 | (131) | Outcome-based spread payments | Governance, process of data collection, payment structure, organisation of payments, outcome correction |
| Espin et al. | 2014 | (132) | Outcome-based agreements | Organisation of data collection, organisation of payments, outcome correction |
| Philipson et al. | 2014 | (133) | Spread payments Outcome-based spread payments | Payment structure, organisation of payments |
| Kornfeld et al. | 2013 | (134) | Outcome-based agreements | Governance |
| Garrison et al. | 2013 | (135) | Outcome-based agreements | Governance, process of data collection, organisation of data collection, organisation of payments, outcome correction |
| Morel et al. | 2013 | (136) | Outcome-based agreements | Process of data collection, organisation of data collection, outcome correction |
| Van Der Meijden et al. | 2013 | (137) | Outcome-based agreements | Process of data collection, organisation of data collection |
| Lage et al. | 2013 | (138) | Outcome-based agreements | Process of data collection, organisation of data collection, outcome correction |
| Ferrario et al. | 2013 | (139) | Outcome-based agreements | Governance, process of data collection, organisation of data collection, organisation of payments |
| Cascade et al. | 2012 | (140) | Outcome-based agreements | Process of data collection, organisation of data collection |
| Goldenberg et al. | 2012 | (141) | Outcome-based agreements | Governance, process of data collection |
| Coulton et al. | 2012 | (142) | Outcome-based agreements | Governance, process of data collection, organisation of data collection, organisation of payments, outcome correction |
| Xoxi et al. | 2012 | (143) | Outcome-based agreements | Governance, process of data collection |
| Towse et al. | 2012 | (144) | Outcome-based agreements | Governance, process of data collection, organisation of data collection, organisation of payments, outcome correction |
| Ferrario et al. | 2011 | (145) | Outcome-based agreements | Governance, organisation of data collection, outcome correction |
| Puig-Peiro et al. | 2011 | (146) | Outcome-based agreements | Governance |
| Jaroslawski et al. | 2011 | (147) | Outcome-based agreements | Governance, process of data collection, organisation of payments, outcome correction |
| Garattini et al. | 2011 | (148) | Outcome-based agreements | Governance, process of data collection, organisation of data collection, organisation of payments, outcome correction |
| Neumann et al. | 2011 | (149) | Outcome-based agreements | Governance, process of data collection, organisation of data collection, outcome correction |
| Loveman et al. | 2011 | (150) | Outcome-based agreements | Governance |
| Menon et al. | 2011 | (151) | Outcome-based agreements | Governance, organisation of data collection, process of data collection, organisation of payments, outcome correction |
| Jaroslawski et al. | 2011 | (152) | Outcome-based agreements | Governance |
| Klemp et al. | 2011 | (153) | Outcome-based agreements | Governance, process of data collection, organisation of data collection |
| McCabe et al. | 2010 | (154) | Outcome-based agreements | Governance, process of data collection, organisation of data collection |
| Towse et al. | 2010 | (155) | Outcome-based agreements | Governance, organisation of data collection |
| Trueman et al. | 2010 | (156) | Outcome-based agreements | Governance, organisation of data collection |
| Stafinski et al. | 2010 | (157) | Outcome-based agreements | Governance, process of data collection, organisation of data collection, outcome correction |
| Carlson et al. | 2010 | (158) | Outcome-based agreements | process of data collection, organisation of data collection |
| Adamski et al. | 2010 | (159) | Outcome-based agreements | Governance, process of data collection, organisation of data collection, organisation of payments, outcome correction |
| Theunissen et al. | 2010 | (160) | Outcome-based agreements | Governance, process of data collection, organisation of data collection |
| Raftery et al. | 2010 | (161) | Outcome-based agreements | Governance, organisation of data collection, outcome correction |
| Williamson et al. | 2010 | (162) | Outcome-based agreements | Governance, organisation of data collection, organisation of payments |
| Menon et al. | 2010 | (163) | Outcome-based agreements | Governance |
| Williamson et al. | 2010 | (164) | Outcome-based agreements | Governance, process of data collection, organisation of data collection, organisation of payments, outcome correction |
| Dankó et al. | 2009 | (165) | Outcome-based agreements | Process of data collection, organisation of data collection, outcome correction |
| Lucas et al. | 2009 | (166) | Outcome-based agreements | Governance, organisation of payments, outcome correction |
| Boggild et al. | 2009 | (167) | Outcome-based agreements | Governance, process of data collection |
| Carlson et al. | 2009 | (168) | Outcome-based agreements | Governance, process of data collection, organisation of data collection, organisation of payments |
| Breckenridge et al. | 2008 | (169) | Outcome-based agreements | Process of data collection |
| Hutton et al. | 2007 | (170) | Outcome-based agreements | Governance, process of data collection, organisation of data collection, organisation of payments |
| Garber et al. | 2007 | (171) | Outcome-based agreements | Governance, process of data collection, outcome correction |
| De Pouvourville et al. | 2006 | (172) | Outcome-based agreements | Governance, process of data collection, organisation of data collection |
| Sudlow et al. | 2003 | (173) | Outcome-based agreements | Governance, organisation of data collection |
| Chapman et al. | 2003 | (174) | Outcome-based agreements | Governance, outcome correction |

# References

1. Kannarkat JT, Good CB, Kelly E, Parekh N. Examining Misaligned Incentives for Payers and Manufacturers in Value-Based Pharmaceutical Contracts. J Manag Care Spec Pharm. 2020;26(1):63-6.

2. Moradi A, Spargo A, Mezzio D, Kaufman S. A discrete choice experiment on payer preferences for innovative payment schemes aimed at financing high-cost, potentially curative therapies. Journal of Managed Care and Specialty Pharmacy. 2019;25:S101-S2.

3. Jönsson B, Hampson G, Michaels J, Towse A, von der Schulenburg JMG, Wong O. Advanced therapy medicinal products and health technology assessment principles and practices for value-based and sustainable healthcare. European Journal of Health Economics. 2019;20(3):427-38.

4. AMCP Partnership Forum: Designing Benefits and Payment Models for Innovative High-Investment Medications. Journal of managed care & specialty pharmacy. 2019;25(2):156-62.

5. Barlow JF, Yang M, Teagarden JR. Are Payers Ready, Willing, and Able to Provide Access to New Durable Gene Therapies? Value in Health. 2019;22(6):642-7.

6. Makady A, van Acker S, Nijmeijer H, de Boer A, Hillege H, Klungel O, et al. Conditional Financing of Drugs in the Netherlands: Past, Present, and Future-Results From Stakeholder Interviews. Value in health : the journal of the International Society for Pharmacoeconomics and Outcomes Research. 2019;22(4):399-407.

7. Jorgensen J, Mungapen L, Kefalas P. Data collection infrastructure for patient outcomes in the UK - opportunities and challenges for cell and gene therapies launching. Journal of market access & health policy. 2019;7(1):1573164.

8. Lorente R, Antonanzas F, Rodriguez-Ibeas R. Implementation of risk-sharing contracts as perceived by Spanish hospital pharmacists. Health Economics Review. 2019;9(1).

9. Makady A, van Veelen A, de Boer A, Hillege H, Klungel OH, Goettsch W. Implementing managed entry agreements in practice: The Dutch reality check. Health Policy. 2019;123(3):267-74.

10. Mundy L, Trowman R, Kearney B. Improving Access to High-Cost Technologies in the Asia Region. International Journal of Technology Assessment in Health Care. 2019;35(3):168-75.

11. Patel N, Deshmukh AV, Ribeiro A. PBI28 Novel Managed Care Strategies can Improve Access to Cellular and Gene Therapy Products. Value in Health. 2019;22:S52-S.

12. Infante K, Madhavan P, Stojanovic I, Kaur S, Nash C. PBI33 Can Innovative Payment Reform be the Solution to Address Reimbursement and Uptake Challenges for CAR-T Therapies? Value in Health. 2019;22:S53-S.

13. Pace J, Kerridge I, Pearson S, Lipworth W. Physicians' attitudes towards accelerated access to medicines. Health economics, policy, and law. 2019:1-16.

14. Macaulay R, Turkstra E. PNS10 Innovative Pharmaceutical Pricing and Reimbursement Schemes in the US, a Cautionary Tales from Abroad. Value in Health. 2019;22:S289-S.

15. Federici C, Reckers-Droog V, Brouwer W, Drummond M. PNS168 Challenges in the Design and Implementation of Coverage with Development Schemes for Medical Devices. Value in Health. 2019;22:S313-S.

16. Goodman C, Villarivera C, Gregor K, van Bavel J. Regulatory, Policy, and Operational Considerations for Outcomes-Based Risk-Sharing Agreements in the U.S. Market: Opportunities for Reform. Journal of managed care & specialty pharmacy. 2019;25(11):1174-81.

17. Antonanzas F, Juarez-Castello C, Lorente R, Rodriguez-Ibeas R. The Use of Risk-Sharing Contracts in Healthcare: Theoretical and Empirical Assessments. Pharmacoeconomics. 2019;37:1469–83.

18. Towse A, Fenwick E. Uncertainty and Cures: Discontinuation, Irreversibility, and Outcomes-Based Payments: What Is Different About a One-Off Treatment? Value in health : the journal of the International Society for Pharmacoeconomics and Outcomes Research. 2019;22(6):677-83.

19. Jorgensen J, Kefalas P. Upgrading the SACT dataset and EBMT registry to enable outcomes-based reimbursement in oncology in England: a gap analysis and top-level cost estimate. Journal of market access & health policy. 2019;7(1):1635842.

20. Mahendraratnam N, Sorenson C, Richardson E, Daniel GW, Buelt L, Westrich K, et al. Value-based arrangements may be more prevalent than assumed. Am J Manag Care. 2019;25(2):70-6.

21. Drummond MF, Neumann PJ, Sullivan SD, Fricke FU, Tunis S, Dabbous O, et al. Analytic Considerations in Applying a General Economic Evaluation Reference Case to Gene Therapy. Value Health. 2019;22(6):661-8.

22. Maes I, Boufraioua H, Schoonaert L, Van Dyck W. Innovative solutions for paradigm changing new therapies – Policy report based on multi-stakeholder round tables. 2019.

23. Cole A, Cubi-Molla P, Pollard J, Sim D, Sullivan R, Sussex J, et al. Making Outcome-based Payment a Reality in the NHS. 2019.

24. Getting Ready: Recommendations for Timely Access to Advanced Therapy Medicinal Products (ATMPs) in Europe. Alliance for Regenerative Medicine; 2019.

25. Payer Perspectives on Financing and Reimbursement of One-time High-cost Durable Treatments. Financing and Reimbursement of Cures in the US (FoCUS); 2019.

26. Wenzl M, Chapman S. Performance-based managed entry agreements for new medicines in OECD countries and EU member states: How they work and possible improvements going forward. OECD Health Working Papers; 2019.

27. Kim H, Comey S, Hausler K, Cook G. A real world example of coverage with evidence development in Australia - ipilimumab for the treatment of metastatic melanoma. Journal of Pharmaceutical Policy and Practice. 2018;11(1):4.

28. Salzman R, Cook F, Hunt T, Malech HL, Reilly P, Foss-Campbell B, et al. Addressing the Value of Gene Therapy and Enhancing Patient Access to Transformative Treatments. Molecular Therapy. 2018;26(12):2717-26.

29. Pham NN, Carlson JJ. Agreement between FDA approved drug label and performance-based risk-sharing arrangement endpoints. Value in Health. 2018;21:S119-S.

30. Robinson MF, Mihalopoulos C, Merlin T, Roughead E. Characteristics of Managed Entry Agreements in Australia. International Journal of Technology Assessment in Health Care. 2018;34(1):46-55.

31. Oren O, Oren M. Cost effectiveness of an outcome based reimbursement model of PCSK9 inhibitors. Journal of the American College of Cardiology. 2018;71(11).

32. Schmetz A, Petrakis I, Khalid JM, Minda K, Agboton C, Rawson K, et al. Darvadstrocel in the Management of Complex Perianal Fistulas: The Role of Patient Registry Data Collection to Support Performance-Based Risk Sharing Agreements. Value in Health. 2018;21:S147-S8.

33. Kefalas P, Ali O, Jorgensen J, Merryfield N, Richardson T, Meads A, et al. Establishing the cost of implementing a performance-based, managed entry agreement for a hypothetical CAR T-cell therapy. Journal of market access & health policy. 2018;6(1):1511679.

34. Hanna E, Toumi M, Dussart C, Borissov B, Dabbous O, Badora K, et al. Funding breakthrough therapies: A systematic review and recommendation. Health Policy. 2018;122(3):217-29.

35. Nevins JF, Colasante W, Rahmati D. PCP13 - Impact of Outcome Based Annuities on Small BioTech Companies. Value in Health. 2018;21:S83-S4.

36. Sachs R, Bagley N, Lakdawalla DN. Innovative Contracting for Pharmaceuticals and Medicaid's Best-Price Rule. Journal of health politics, policy and law. 2018;43(1):5-18.

37. Maskineh C, Nasser SC. Managed Entry Agreements for Pharmaceutical Products in Middle East and North African Countries: Payer and Manufacturer Experience and Outlook. Value in health regional issues. 2018;16:33-8.

38. Bouvy JC, Sapede C, Garner S. Managed Entry Agreements for Pharmaceuticals in the Context of Adaptive Pathways in Europe. Frontiers in pharmacology. 2018;9:280.

39. Lidonnici D, Lanati EP, Niedecker S, Isernia M. New drugs approval in Italy: Updated analysis of the applied negotiation conditions 2015-2017. Value in Health. 2018;21:S92-S.

40. Richardson TD, Ling C. Patient Access To New Medicines; The Changing Landscape. Value in Health. 2018;21:S83-S.

41. Brown JD, Sheer R, Pasquale M, Sudharshan L, Axelsen K, Subedi P, et al. Payer and Pharmaceutical Manufacturer Considerations for Outcomes-Based Agreements in the United States. Value in health : the journal of the International Society for Pharmacoeconomics and Outcomes Research. 2018;21(1):33-40.

42. Sandhu AT, Heidenreich PA. Pearls and pitfalls of performance-linked reimbursement plans for novel drugs: The case of sacubitril-valsartan. Circulation: Cardiovascular Quality and Outcomes. 2018;11.

43. Antonanzas F, Rodriguez-Ibeas R, Juarez-Castello CA. Personalized Medicine and Pay for Performance: Should Pharmaceutical Firms be Fully Penalized when Treatment Fails? Pharmacoeconomics. 2018;36(7):733-43.

44. Goncalves FR, Santos S, Silva C, Sousa G. Risk-sharing agreements, present and future. Ecancermedicalscience. 2018;12:823.

45. Goldenberg D, Livermore R, Moore B, Katiyar J. The current landscape and future of outcomes-based contracting with commercial healthcare payers in the US. Value in Health. 2018;21:S66-S7.

46. Duhig AM, Saha S, Smith S, Kaufman S, Hughes J. The Current Status of Outcomes-Based Contracting for Manufacturers and Payers: An AMCP Membership Survey. Journal of managed care & specialty pharmacy. 2018;24(5):410-5.

47. Urbinati D, Cioni L, Rova A. The Evolution of Standard Monitoring Registries in the Italian Market Value in Health. 2018;21:S210-S.

48. Carr D, Ermacora P, Nicholson T, Grosvenor A. The Future of Value-Based Contracting for High-Cost Innovative Therapies: Global Lessons From Europe and The US. Value in Health. 2018;21:S211-S.

49. Jorgensen J, Servos S, Kefalas P. The potential price and access implications of the cost-utility and budget impact methodologies applied by NICE in England and ICER in the US for a novel gene therapy in Parkinson's disease. Journal of market access & health policy. 2018;6(1):1500419.

50. Danzon PM. Affordability Challenges to Value-Based Pricing: Mass Diseases, Orphan Diseases, and Cures. Value Health. 2018;21(3):252-7.

51. Faulkner E, Werner MJ, Slocomb T, Han D. Ensuring patient access to regenerative and advanced therapies in managed care: how do we get there? Journal of Managed Care Medicine. 2018.

52. Senior M. Rollout of high-priced cell and gene therapies forces payer rethink. Nat Biotechnol. 2018;36(4):291-2.

53. Menner J, Lewandowska PK. PHP354 - The Impact of Data Privacy Regulations on Drug Utilization Data Sharing for Innovative Pricing Arrangements. Value in Health. 2018;21:S211.

54. Stirnadel-Farrant H, Kudari M, Garman N, Imrie J, Chopra B, Giannelli S, et al. Gene therapy in rare diseases: the benefits and challenges of developing a patient-centric registry for Strimvelis in ADA-SCID. Orphanet J Rare Dis. 2018;13(1):49.

55. Tuffaha HW, Scuffham PA. The Australian Managed Entry Scheme: Are We Getting it Right? Pharmacoeconomics. 2018;36(5):555-65.

56. Mailankody S, Bach PB. Money-Back Guarantees for Expensive Drugs: Wolf's Clothing but a Sheep Underneath. Annals of Internal Medicine. 2018;168(12):888-9.

57. Innovative Payment Models for High-Cost Innovative Medicines. European Commission: Expert Panel on effective ways of investing in Health (EXPH); 2018.

58. The missing piece - How EY’s Health Outcomes Platform can create “triple wins” through outcomes-based contracting. Ernst & Young; 2018.

59. Passing fad or game-changer? Outcomes-based contracting in life sciences. Ernst & Young; 2018.

60. Precision Financing Solutions for Durable / Potentially Curative Therapies. Financing and Reimbursement of Cures in the US (FoCUS); 2019.

61. AMCP Partnership Forum: Advancing Value-Based Contracting. journal of managed care & specialty pharmacy. 2017;23(11):1096-102.

62. Jorgensen J, Kefalas P. Annuity payments can increase patient access to innovative cell and gene therapies under England's net budget impact test. Journal of market access & health policy. 2017;5(1):1355203.

63. Holleman MS, Van Der Linden N, Uyl-De Groot CA. Determining the comparative value of outcome-based money-back guarantee scenarios in non-small cell lung cancer using real-world data. Value in Health. 2017;20(9):A466-A.

64. Calabrese MJ, Cooke CE, Watson K, de Bittner MR. Emerging roles for pharmacists in performance-based risk-sharing arrangements. American Journal of Health-System Pharmacy. 2017;74(13):1007-12.

65. Macaulay R, Hettle R. Innovative pricing and reimbursement schemes-the what , why, which, and how. Value in Health. 2017;20(9):A403-A4.

66. Pauwels K, Huys I, Vogler S, Casteels M, Simoens S. Managed entry agreements for oncology drugs: Lessons from the European experience to inform the future. Frontiers in Pharmacology. 2017;8(APR):171.

67. Nazareth T, Ko JJ, Sasane R, Frois C, Carpenter S, Demean S, et al. Outcomes-Based Contracting Experience: Research Findings from U.S. and European Stakeholders. Journal of managed care & specialty pharmacy. 2017;23(10):1018-26.

68. Spark YM, Sear R, Hutchings A. Paying for gene therapies: Approaching a sustainable solution. Value in Health. 2017;20(9):A685-A.

69. Yu JS, Chin L, Oh J, Farias J. Performance-based risk-sharing arrangements for pharmaceutical products in the United States: A systematic review. Journal of Managed Care and Specialty Pharmacy. 2017;23(10):1028-40.

70. Carlson JJ, Chen S, Garrison LP, Jr. Performance-Based Risk-Sharing Arrangements: An Updated International Review. Pharmacoeconomics. 2017;35(10):1063-72.

71. Goble JA, Ung B, van Boemmel-Wegmann S, Navarro RP, Parece A. Performance-Based Risk-Sharing Arrangements: U.S. Payer Experience. Journal of managed care & specialty pharmacy. 2017;23(10):1042-52.

72. Hettle R, Corbett M, Hinde S, Hodgson R, Jones-Diette J, Woolacott N, et al. The assessment and appraisal of regenerative medicines and cell therapy products: An exploration of methods for review, economic evaluation and appraisal. Health Technology Assessment. 2017;21(7):1-204.

73. van de Wetering EJ, van Exel J, Brouwer WBF. The Challenge of Conditional Reimbursement: Stopping Reimbursement Can Be More Difficult Than Not Starting in the First Place! Value in health : the journal of the International Society for Pharmacoeconomics and Outcomes Research. 2017;20(1):118-25.

74. Toumi M, Jarosławski S, Sawada T, Kornfeld A. The Use of Surrogate and Patient-Relevant Endpoints in Outcomes-Based Market Access Agreements: Current Debate. Applied Health Economics and Health Policy. 2017;15(1):5-11.

75. Yeung K, Li M, Carlson JJ. Using performance-based risk-sharing arrangements to address uncertainty in indication-based pricing. Journal of Managed Care and Specialty Pharmacy. 2017;23(10):1010-5.

76. Yeung K, Suh K, Basu A, Garrison LP, Bansal A, Carlson JJ. Paying for Cures: How Can We Afford It? Managed Care Pharmacy Stakeholder Perceptions of Policy Options to Address Affordability of Prescription Drugs. J Manag Care Spec Pharm. 2017;23(10):1084-90.

77. Clopes A, Gasol M, Cajal R, Segu L, Crespo R, Mora R, et al. Financial consequences of a payment-by-results scheme in Catalonia: gefitinib in advanced EGFR-mutation positive non-small-cell lung cancer. J Med Econ. 2017;20(1):1-7.

78. Hampson G, Towse A, Pearson SD, Dreitlein WB, Henshall C. Gene therapy: Evidence, value and affordability in the US health care system. Journal of Comparative Effectiveness Research. 2018;7(1):15-28.

79. Kanavos P, Ferrario A, Tafuri G, Siviero P. Managing Risk and Uncertainty in Health Technology Introduction: The Role of Managed Entry Agreements. Global Policy. 2017;8(S2):84-92.

80. Schaffer SK, Messner D, Mestre-Ferrandiz J, Tambor E, Towse A. Paying for Cures: Perspectives on Solutions to the "Affordability Issue". Value Health. 2018;21(3):276-9.

81. Kazi DS, Penko J, Coxson PG, Moran AE, Ollendorf DA, Tice JA, et al. Updated Cost-effectiveness Analysis of PCSK9 Inhibitors Based on the Results of the FOURIER Trial. JAMA. 2017;318(8):748-50.

82. Developing a Path to Value-Based Payment for Medical Products: Value-Based Payment Advisory Group Kick-Off Meeting. Duke-Margolis Center for Health Policy Conference Center; 2017.

83. Annemans L, Pani L. Dynamic outcomes based approaches to pricing and reimbursement of innovative medicines. 2017. p. 1-12.

84. Gerkens S, Neyt M, San Miguel L, Vinck I, Thiry N, Cleemput I. How to improve the Belgian process for managed entry agreements? An analysis of the Belgian and international experience. Belgian Health Care Knowledge Centre (KCE); 2017.

85. Slocomb T, Werner M, Haack T, Valluri S, Rader B. New Payment And Financing Models For Curative Regenerative Medicines. In Vivo. 2017.

86. Launching into value: Pharma’s quest to align drug prices with outcomes. PWC Health Research Institute; 2017.

87. Fox J, Watrous M. Overcoming Challenges Of Outcomes-Based Contracting For Pharmaceuticals: Early Lessons From The Genentech--Priority Health Pilot. Health Affairs; 2017.

88. Seeley E, Kesselheim AS. Outcomes-Based Pharmaceutical Contracts: An Answer to High U.S. Drug Spending? : The Commonwealth Fund; 2017.

89. Moving Forward on Value-Based Contracting for Biopharmaceuticals. Network for Excellence in Health Innovation (NEHI); 2017.

90. Marsden G, Towse A, Pearson SD, Dreitlein B, Henshall C. Gene Therapy: Understanding the Science, Assessing the Evidence, and Paying for Value. 2016 ICER Membership Policy Summit; 2017.

91. Carroll E, Truglio A. A risk-benefit analysis of key performance-based risk sharing agreements (PBRSAs) in the EU and US (2002-2016). Value in Health. 2016;19(7):A500-A.

92. Proach J, Black J, Cromer K, Winters D. Exploring alternative payment methodologies for innovative, expensive treatments: What is needed to trigger a change from the current upfront payment system? Value in Health. 2016;19(3):A258-A.

93. Carr DR, Bradshaw SE. Gene therapies: The challenge of super-high-cost treatments and how to pay for them. Regenerative Medicine. 2016;11(4):381-93.

94. Barlas S. Health Plans and Drug Companies Dip Their Toes Into Value-Based Pricing: The Pressure Is on P&T Committees to Monitor Utilization. P & T : a peer-reviewed journal for formulary management. 2016;41(1):39-53.

95. Pouwels XG, Grutters JP, Bindels JA, Ramaekers BL, Joore MA. Missed opportunities in the netherlands: The negligence of non-statistical uncertainty in CED policy. Value in Health. 2016;19(7):A750-A.

96. Malik NN. Pay-for-performance pricing for a breakthrough heart drug: learnings for cell and gene therapies. 2016. p. 225-7.

97. Polimeni G, Isgrò V, Aiello A, D'Ausilio A, D'Addetta, Cuzzocrea S, et al. Role of clinical pharmacist in optimizing reimbursement originating from performance-based risk-sharing arrangements: The experience of the university hospital “G. Martino” from Messina, Italy. Value in Health. 2016;19(7):A756-A.

98. Van De Vijver I, Quanten A, Knappenberg V, Arickx F, De Ridder R. Success and failure of straightforward versus sophisticated managed entry agreements. Value in Health. 2016;19(7):A499-A.

99. Thompson M, Henshall C, Garrison LP, Griffin AD, Coyle D, Long S, et al. Targeting improved patient outcomes using innovative product listing agreements: A survey of canadian and international key opinion leaders. ClinicoEconomics and Outcomes Research. 2016;8:427-33.

100. Kiernan F. The future of pharmacoeconomic policy - does value-based pricing really have a role? Journal of Pharmaceutical Health Services Research. 2016;7(1):5-9.

101. Barlas S. Value Purchasing Programs Make Plodding Progress: Drug Manufacturers Question Some Pay-for-Performance Methodologies. P & T : a peer-reviewed journal for formulary management. 2016;41(9):562-89.

102. Montazerhodjat V, Weinstock DM, Lo AW. Buying cures versus renting health: Financing health care with consumer loans. Science Translational Medicine. 2016;8(327):327ps6.

103. Abou-El-Enein M, Elsanhoury A, Reinke P. Overcoming Challenges Facing Advanced Therapies in the EU Market. Cell Stem Cell. 2016;19(3):293-7.

104. Faulkner SD, Lee M, Qin D, Morrell L, Xoxi E, Sammarco A, et al. Pricing and reimbursement experiences and insights in the European Union and the United States: Lessons learned to approach adaptive payer pathways. Clin Pharmacol Ther. 2016;100(6):730-42.

105. Pritchett L, Wiesinger A, Faria-Billinton E, Brown A, Murray G, Stoor L, et al. A review of guidelines and approaches to performance-based risk-sharing agreements across the UK, Italy and the Netherlands. Value in Health. 2015;18(7):A568-A.

106. De Rosa M, Martini N, Ortali M, Esposito I, Roncadori A. An innovative cloud-based platform for implementing performance-based risk-sharing arrangements (PBRSAs) in oncology settings. Value in Health. 2015;18(3):A273-A.

107. Lewis JRR, Kerridge I, Lipworth W, J.R.R L, I K, W L. Coverage with evidence development and managed entry in the funding of personalized medicine: Practical and ethical challenges for oncology. Journal of Clinical Oncology. 2015;33(34):4112-7.

108. Navarria A, Drago V, Gozzo L, Longo L, Mansueto S, Pignataro G, et al. Do the current performance-based schemes in Italy really work? "Success fee": A novel measure for cost-containment of drug expenditure. Value in Health. 2015;18(1):131-6.

109. Mohseninejad L, Van Gils C, Uyl-De Groot CA, Buskens E, Feenstra T. Evaluation of patient registries supporting reimbursement decisions: The case of oxaliplatin for treatment of stage III colon cancer. Value in Health. 2015;18(1):84-90.

110. Lucas F, Wong I. Payer vs. industry views on managed entry agreements. Value in Health. 2015;18(7):A568-A9.

111. Garrison LP, Jr., Carlson JJ, Bajaj PS, Towse A, Neumann PJ, Sullivan SD, et al. Private sector risk-sharing agreements in the United States: trends, barriers, and prospects. The American journal of managed care. 2015;21(9):632-40.

112. Kocsis T, Papp E, Nemeth B, Juhasz J. The cost of treatment of the new antiviral therapies against the hepatitis C virus. Value in Health. 2015;18(7):A689-A.

113. Drummond M. When do performance-based risk-sharing arrangements make sense? European Journal of Health Economics. 2015;16(6):569-71.

114. Kleinke JD, McGee N. Breaking the Bank: Three Financing Models for Addressing the Drug Innovation Cost Crisis. American health & drug benefits. 2015;8(3):118-26.

115. Garattini L, Curto A, van de Vooren K. Italian risk-sharing agreements on drugs: are they worthwhile? Eur J Health Econ. 2015;16(1):1-3.

116. Montilla S, Xoxi E, Russo P, Cicchetti A, Pani L. Monitoring Registries at Italian Medicines Agency: Fostering Access, Guaranteeing Sustainability. Int J Technol Assess Health Care. 2015;31(4):210-3.

117. Lu CY, Lupton C, Rakowsky S, Babar Z-U-D, Ross-Degnan D, Wagner AK. Patient access schemes in Asia-pacific markets: current experience and future potential. Journal of pharmaceutical policy and practice. 2015;8(1):6.

118. Jorgensen J, Kefalas P. Reimbursement of licensed cell and gene therapies across the major European healthcare markets. J Mark Access Health Policy. 2015;3.

119. Touchot N, Flume M. The payers' perspective on gene therapies. Nat Biotechnol. 2015;33(9):902-4.

120. Tuna E, Tatar M, Ergin G, Senturk A, Atikeler K. Availability of risk sharing agreements in the turkish pharmaceutical sector. Value in Health. 2014;17(7):A415-A.

121. Li CM, Risebrough NA, Hux M. Coverage with evidence development activities around the world: An environment scan. Value in Health. 2014;17(7):A449-A.

122. Carlson JJ, Gries K, Sullivan SD, Garrison L. Current status and trends in performance-based schemes between health care payers and manufacturers. Value in Health. 2011;14(7):A359-A60.

123. Launois R, Navarrete LF, Ethgen O, Le Moine J-G, Gatsinga R. Health economic value of an innovation: delimiting the scope and framework of future market entry agreements. Journal of market access & health policy. 2014;2.

124. Vitry A, Roughead E. Managed entry agreements for pharmaceuticals in Australia. Health Policy. 2014;117(3):345-52.

125. Gibson SG, Lemmens T. Niche markets and evidence assessment in transition: a critical review of proposed drug reforms. Medical law review. 2014;22(2):200-20.

126. Franken MG, Gaultney JG, Blommestein HM, Huijgens PC, Sonneveld P, Redekop WK, et al. Policymaker, please consider your needs carefully: does outcomes research in relapsed or refractory multiple myeloma reduce policymaker uncertainty regarding value for money of bortezomib? Value in health : the journal of the International Society for Pharmacoeconomics and Outcomes Research. 2014;17(2):245-53.

127. Bibeau J, Savoie M, Lachaine J. Real-world clinical evidence development: An analysis of relevant international models for the potential implementation of such a program in quebec. Value in Health. 2014;17(3):A17-A8.

128. Edlin R, Hall P, Wallner K, McCabe C. Sharing risk between payer and provider by leasing health technologies: An affordable and effective reimbursement strategy for innovative technologies? Value in Health. 2014;17(4):438-44.

129. Gottlieb S, Carino T. Establishing new payment provisions for the high cost of curing disease. AEI Research; 2014.

130. Basu A. Financing cures in the United States. Expert Rev Pharmacoecon Outcomes Res. 2015;15(1):1-4.

131. Brennan TA, Wilson JM. The special case of gene therapy pricing. Nat Biotechnol. 2014;32(9):874-6.

132. Espin J, Rovira J, Garcia L. Experiences and Impact of European Risk-Sharing Schemes Focusing on Oncology Medicines. EMINet; 2011.

133. Philipson T. Medical Breakthroughs And Credit Markets: Forbes; 2014 [Available from: <https://www.forbes.com/sites/tomasphilipson/2014/07/09/medical-breakthroughs-and-credit-markets/#1014decf701c>.

134. Kornfeld A, Schroeder M, Toumi T. Coverage with evidence development in Sweden - Formality or effective way to reduce uncertainty? Value in Health. 2013;16(7):A487-A8.

135. Garrison LP, Jr., Towse A, Briggs A, de Pouvourville G, Grueger J, Mohr PE, et al. Performance-based risk-sharing arrangements - Good practices for design, implementation, and evaluation: Report of the ISPOR good practices for performance-based risk-sharing arrangements task force. Value in Health. 2013;16(5):703-19.

136. Morel T, Arickx F, Befrits G, Siviero P, van der Meijden C, Xoxi E, et al. Reconciling uncertainty of costs and outcomes with the need for access to orphan medicinal products: A comparative study of managed entry agreements across seven European countries. Orphanet Journal of Rare Diseases. 2013;8(1):198.

137. Van Der Meijden CMJ, Streuper C, Goettsch W. The first experiences with reassessments and appraisals of conditionally approved expensive and orphan drugs in the Netherlands (2006-2013). Value in Health. 2013;16(7):A376-A.

138. Lage MJ, Carroll CA, Fairman KA. Using observational analysis of multiple sclerosis relapse to design outcomes-based contracts for disease-modifying drugs: a feasibility assessment. Journal of medical economics. 2013;16(9):1146-53.

139. Ferrario A, Kanavos P. Managed entry agreements for pharmaceuticals: The European experience. EMINet; 2013.

140. Cascade E, Andreykiv M, Istas A. Payer and patient perceptions of medication value and the role of patient-reported outcomes. Value in Health. 2012;15(7):A481-A2.

141. Goldenberg D, Bachman EM. The future of alternative pricing/risk-sharing agreements in the United States. Value in Health. 2012;15(4):A30-A.

142. Coulton L, Annemans L, Carter R, Herrera MB, Thabrany H, Lim J, et al. Outcomes-based Risk-sharing Schemes: Is There a Potential Role in the Asia-Pacific Markets? Health Outcomes Res Med. 2012;3(4):e205-e19.

143. Xoxi E, Tomino C, de Nigro L, Pani L. The Italian post-marketing registries. Pharmaceutical Programming. 2012;5(1-2):57-60.

144. Towse A, Garrison L, Puig-Peiro R. The Use of Pay-for-Performance for Drugs: Can It Improve Incentives for Innovation? : Office of Health Economics & University of Washington; 2012.

145. Ferrario A, Nicod E, Kanavos P. Evidence on the impact of managed entries on payers, patients, manufacturers, and health care workers. Value in Health. 2011;14(7):A360-A1.

146. Puig-Peiró R, Mestre-Ferrandiz J, Sussex J, Towse A. Literature review on patient access schemes, flexible pricing schemes and risk sharing agreements for medicines. Value in Health. 2011;14(7):A243-A.

147. Jaroslawski S, Toumi M. Market Access Agreements for pharmaceuticals in Europe: diversity of approaches and underlying concepts. BMC health services research. 2011;11:259.

148. Garattini L, Casadei G. Risk sharing agreements: What lessons from Italy? International Journal of Technology Assessment in Health Care. 2011;27(2):169-72.

149. Neumann PJ, Chambers JD, Simon F, Meckley LM. Risk-sharing arrangements that link payment for drugs to health outcomes are proving hard to implement. Health Affairs. 2011;30(12):2329-37.

150. Loveman CM, Mallinson M, Areteou T, White R. Value based pricing: What is the future for patient access schemes? Value in Health. 2011;14(3):A30-A.

151. Menon D, Stafinski T, Nardelli A, Jackson T, Jhamandas J. Access with evidence development: an approach to introducing promising new technologies into healthcare. Healthc Manage Forum. 2011;24(2):42-56.

152. Jaroslawski S, Toumi M. Design of patient access schemes in the UK: influence of health technology assessment by the National Institute for Health and Clinical Excellence. Appl Health Econ Health Policy. 2011;9(4):209-15.

153. Klemp M, Fronsdal KB, Facey K. What principles should govern the use of managed entry agreements? Int J Technol Assess Health Care. 2011;27(1):77-83.

154. McCabe CJ, Stafinski T, Edlin R, Menon D. Access with evidence development schemes: A framework for description and evaluation. Pharmacoeconomics. 2010;28(2):143-52.

155. Towse A, Garrison LP, Jr. Can't get no satisfaction? Will pay for performance help?: Toward an economic framework for understanding performance-based risk-sharing agreements for innovative medical products. Pharmacoeconomics. 2010;28(2):93-102.

156. Trueman P, Grainger DL, Downs KE. Coverage with evidence development: Applications and issues. International Journal of Technology Assessment in Health Care. 2010;26(1):79-85.

157. Stafinski T, McCabe C, Menon D. Funding the unfundable: Mechanisms for managing uncertainty in decisions on the introduction of new and innovative technologies into healthcare systems. Pharmacoeconomics. 2010;28(2):113-42.

158. Carlson JJ, Sullivan SD, Garrison LP, Neumann PJ, Veenstra DL. Linking payment to health outcomes: A taxonomy and examination of performance-based reimbursement schemes between healthcare payers and manufacturers. Health Policy. 2010;96(3):179-90.

159. Adamski J, Godman B, Ofierska-Sujkowska G, Osinska B, Herholz H, Wendykowska K, et al. Risk sharing arrangements for pharmaceuticals: potential considerations and recommendations for European payers. BMC health services research. 2010;10:153.

160. Theunissen KA, Delwel G, Evers SM, Goettsch W, Severens H, Hoomans T. Successful implementation of coverage with evidence development schemes: Practical experiences in several western jurisdictions. Value in Health. 2010;13(7):A420-A.

161. Raftery J. Multiple sclerosis risk sharing scheme: a costly failure. BMJ. 2010;340:c1672.

162. Williamson S. Patient access schemes for high-cost cancer medicines. Lancet Oncol. 2010;11(2):111-2.

163. Menon D, McCabe CJ, Stafinski T, Edlin R. Principles of design of access with evidence development approaches: a consensus statement from the Banff Summit. Pharmacoeconomics. 2010;28(2):109-11.

164. Williamson S, Thomson D. A report into the uptake of patient access schemes in the NHS. Clinical Pharmacist. 2010;2.

165. Dankó D, Molnár MP, Berta G. Designing and implementing outcome-based reimbursement schemes: Experience from Hungary. Value in Health. 2009;12(7):A229-A.

166. Lucas F, Easley C, Jackson G. The usefulness and challenges of patient cess (risk sharing) schemes in the UK. Value in Health. 2009;12(7):A243-A.

167. Boggild M, Palace J, Barton P, Ben-Shlomo Y, Bregenzer T, Dobson C, et al. Multiple sclerosis risk sharing scheme: two year results of clinical cohort study with historical comparator. BMJ. 2009;339:b4677.

168. Carlson JJ, Garrison LP, Jr., Sullivan SD. Paying for outcomes: innovative coverage and reimbursement schemes for pharmaceuticals. J Manag Care Pharm. 2009;15(8):683-7.

169. Breckenridge A, Walley T. Risk Sharing and Payment by Results. Clin Pharmacol Ther. 2008;83(5):666-7.

170. Hutton J, Trueman P, Henshall C. Coverage with evidence development: an examination of conceptual and policy issues. Int J Technol Assess Health Care. 2007;23(4):425-32.

171. Garber AM, McClellan MB. Satisfaction guaranteed--"payment by results" for biologic agents. N Engl J Med. 2007;357(16):1575-7.

172. de Pouvourville G. Risk-sharing agreements for innovative drugs: a new solution to old problems? Eur J Health Econ. 2006;7(3):155-7.

173. Sudlow CL, Counsell CE. Problems with UK government's risk sharing scheme for assessing drugs for multiple sclerosis. BMJ. 2003;326(7385):388-92.

174. Chapman S, Reeve E, Rajaratnam G, Neary R. Setting up an outcomes guarantee for pharmaceuticals: new approach to risk sharing in primary care. BMJ. 2003;326(7391):707-9.
